# Cognitive impairment in people with schizophrenia: an umbrella review

**DOI:** 10.1007/s00406-022-01416-6

**Published:** 2022-05-28

**Authors:** Yohannes Gebreegziabhere, Kassahun Habatmu, Awoke Mihretu, Matteo Cella, Atalay Alem

**Affiliations:** 1 Department of Nursing, College of Health Sciences, Debre Berhan University, Debre Berhan, Ethiopia; 2 Department of Psychiatry, College of Health Sciences, Addis Ababa University, Addis Ababa, Ethiopia; 3 School of Psychology, College of Education and Behavioral Studies, Addis Ababa University, Addis Ababa, Ethiopia; 4 Department of Psychology, Institute of Psychiatry, Psychology and Neuroscience, King’s College London, London, England UK

**Keywords:** Cognitive impairment, Domains impaired, Determinants, Schizophrenia

## Abstract

Cognitive impairment is one of the core symptoms of schizophrenia. Quite a number of systematic reviews were published related to cognitive impairment in people with schizophrenia (PWS). This umbrella review, therefore, aimed at reviewing and synthesizing the findings of systematic reviews related to domains of cognition impaired and associated factors in PWS. We searched four electronic databases. Data related to domains, occurrence, and associated factors of cognitive impairment in PWS were extracted. The quality of all eligible systematic reviews was assessed using A MeaSurement Tool to Assess methodological quality of systematic Review (AMSTAR) tool. Results are summarized and presented in a narrative form. We identified 63 systematic reviews fulfilling the eligibility criteria. The included reviews showed that PWS had lower cognitive functioning compared to both healthy controls and people with affective disorders. Similar findings were reported among psychotropic free cases and people with first episode psychosis. Greater impairment of cognition was reported in processing speed, verbal memory, and working memory domains. Greater cognitive impairment was reported to be associated with worse functionality and poor insight. Cognitive impairment was also reported to be associated with childhood trauma and aggressive behaviour. According to our quality assessment, the majority of the reviews had moderate quality. We were able to find a good number of systematic reviews on cognitive impairment in PWS. The reviews showed that PWS had higher impairment in different cognitive domains compared to healthy controls and people with affective disorders. Impairment in domains of memory and processing speed were reported frequently.

**Supplementary Information:**

The online version contains supplementary material available at 10.1007/s00406-022-01416-6.

## Introduction

Cognitive function is a mental process which involves several intellectual abilities such as perception, reasoning, and remembering, while cognitive impairment is the malfunction of cognition or intellectual abilities [1]. Cognitive impairment in people with schizophrenia (PWS) can be considered as part of core symptoms of the disorder because: (1) almost all (98%) PWS showed cognitive decrement compared to their premorbid state [2], (2) PWS showed a broader domains of cognitive impairment with varying severity per domain [3], and (3) cognitive impairment is seen in PWS who are not taking anti-psychotic drugs [4].

Cognitive impairment in PWS was recognized from as early as the time of Emil Kraepelin, where Kraepelin named the disorder as dementia praecox to mean early-onset dementia (although due attention has not been given to it) [5]. However, different groups are now working to improve cognitive impairment in PWS as part of the intervention for the problem [6, 7]. One of these groups is the Measurement And Treatment Research to Improve Cognition in Schizophrenia (MATRICS) initiative [6]. The MATRICS initiative, through review of factor analytical studies and consensus, identified seven domains of cognition which are more affected in PWS [8]. Brief description of each of these domains is given below (Table 1).

**Table 1:** Brief description of cognitive domains which are more affected and commonly reported in people with schizophrenia

Domain	Definition	Citation
Speed of processing	The speed in which information is processed and encoded to give meaning about the outside world	(Bowie and Harvey, 2005 [76]; Braff, 1993 [77])
Attention/concentration	Attention is the ability to detect and focus on relevant stimuli, and ignore irrelevant stimuli. Concentration is the ability to sustain attention	
Working memory	The ability to store and manipulate information for short period of time	
Verbal learning and memory	The ability to learn and remember from verbal stimuli	
Visual learning and memory	The ability to learn and remember from visual stimuli	
Reasoning and problem solving	The ability to solve problem, think abstractly, and coordinate other cognitive domains	
Social cognition	The ability to perceive and make sense of the surrounding, which includes four interrelated sub-domains: (1) Theory of mind; the ability to make inference about other beliefs, dispositions, and intentions (2) Emotion (perception and) processing; is the ability of a person related to identification, facilitation, and managing emotion (3) Social perception and social knowledge; Social perception is the ability to use cues to identify social context, roles, and rules. Whereas, Social knowledge is the ability to identify context and rules to apply in an identified specific social context (4) Attributional bias; it is the kind of inference that a person makes about the causes of a positive and negative event in their life	(Kayman and Goldstein, 2012 [78])

A large number of articles are published on cognitive impairment in PWS. A simple hit of “Cognition AND Schizophrenia” in PubMed yields around 30,000 articles. Consequently, quite a number of systematic reviews have been published on the subject. Although there are numerous systematic reviews on the magnitude of cognitive domains impaired and associated factors in PWS, we found only one published umbrella review on the subject [9]. However, this umbrella review has not addressed factors associated with cognitive impairment; and it only focused on the difference in the magnitude of cognitive impairment between PWS and people with bipolar disorder.

The Diagnostic and Statistical Manual of mental disorders (DSM) [5] and the International Classification of Diseases (ICD) [10] have not, to date, considered cognitive impairment in their diagnostic criteria for schizophrenia. Whereas, cognitive impairment seems to be one of the core symptoms of this illness. In this umbrella review, we aimed to explore the evidence that cognitive impairment maybe a common problem in PWS by examining findings from systematic reviews and meta-analysis related to magnitude of cognitive domains impaired and associated factors in PWS. This umbrella review might help clinicians and experts in the area to have a comprehensive understanding of domains of cognition affected, and related factors in PWS. Additionally, this review would inform future researchers regarding which factors need to be considered while planning studies involving cognitive impairment in PWS. This umbrella review, therefore, aimed at synthesizing the evidence on magnitude of cognitive domains impaired and associated factors in PWS from systematic review studies.

## Methods

We followed the Preferred Reporting Items for Systematic Reviews and Meta-Analyses (PRISMA) guideline to identify, search, extract articles, and report this umbrella review [11].

### Databases searched

We searched major databases, including PubMed, Embase, PsycINFO, and Global Index Medicus from the date of inception of each database until July 05, 2018. An update search from the same databases was conducted on 13th August 2020. Google Scholar was used for forward and backward searching.

### Search strategy

We used the following three key words: schizophrenia, Cognition, and Magnitude/Associated factors. Free terms and controlled vocabulary terms were used for those three big terms. We combined those three big terms with the Boolean term “AND”. The term systematic review was not included in the initial search to allow collection of systematic reviews that did not mention systematic review in their title or abstract, however, we later filtered each databases for review or systematic review or meta-analysis or other related terms according to the filter method of the database. For the complete search strategy, see online resource 1. To increase our chance of capturing all systematic reviews about the magnitude and associated factors of cognitive impairment in PWS, we conducted a forward and backward search.

### Eligibility criteria

This umbrella review considered reviews that aimed at assessing domains of cognitive impairment and associated factors among adult PWS aged 18 years and older. Diagnoses needed to have been confirmed using either DSM [5], ICD [10], or other recognized diagnostic criteria. For studies with mixed populations at least 50% of the participants should be PWS.

We included any systematic reviews or meta-analysis which addressed concepts related to magnitude and/or associated factors of at least one full domain of cognition in PWS. If the review was about sub-domains of a single domain then that review was excluded.

We only included systematic review studies. In this review, systematic review was operationalized as any review which had a search term, searched at least one database, had eligibility criteria to include studies, and reported the findings systematically [12]. We employed such a stringent definition of systematic review, because there were numerous unstructured reviews, expert comments, editorials, overviews, guidelines, and other non-systematic reviews on the subject.

No restriction was employed in terms of settings of the systematic reviews. However, systematic reviews published only in English were included into this umbrella review.

### Full-text identification process

We merged the articles we found from the databases and removed duplicates. The first author (YG) screened each article for eligibility using their title and abstract, followed by full-text screening. While, another author (AM) screened 10% of the articles and we found good agreement. Same procedure was employed in screening articles obtained through forward and backward search.

### Data extraction

The first author (YG) extracted data from the included articles using a data extraction tool developed a priori. Another author (AM) checked a randomly selected articles for correct extraction using the same extraction tool. The extraction tool was developed referring to previous published systematic reviews, and the requirements for quality assessment in consultation with the other co-authors. This was followed by piloting it on two articles (the data extraction template is in online resource 2). The core components of the data extraction tool include authors’ detail, databases searched, number of included studies, total number of participants included, cognitive domains addressed, and findings about cognitive impairment and associated factors.

### Risk of bias/quality assessment

The first author (YG) and another author (AM) assessed the quality of each review using a critical appraisal form designed for evaluating the methodological quality of systematic reviews: a measurement tool to assess methodological quality of systematic Review (AMSTAR) [13]. AMSTAR has eleven items each to be scored as one for “yes” and zero for “no”. The eleven items focused on the presence of a prior registration of the protocol, study identification process, quality assessment section, and other steps of conducting a review. A score of 8 or more is considered as a good-quality review, a score of 4 to 7 a moderate quality, and a score below 4 a poor-quality review.

### Data synthesis

We used a narrative synthesis to report the findings in this review. A narrative synthesis approach was selected as this approach is more appropriate to summarize studies with heterogeneous outcomes. For each systematic review included, we have reported the databases searched, number of included studies, total number of participants in each group (if more than one group was involved), specific cognitive domains evaluated, and findings related to magnitude and associated factors of cognitive impairment. We have also reported methodological qualities of each review included. We have presented the findings considering the methodological quality of the reviews included.

## Results

### Study characteristics

**Fig. 1:**
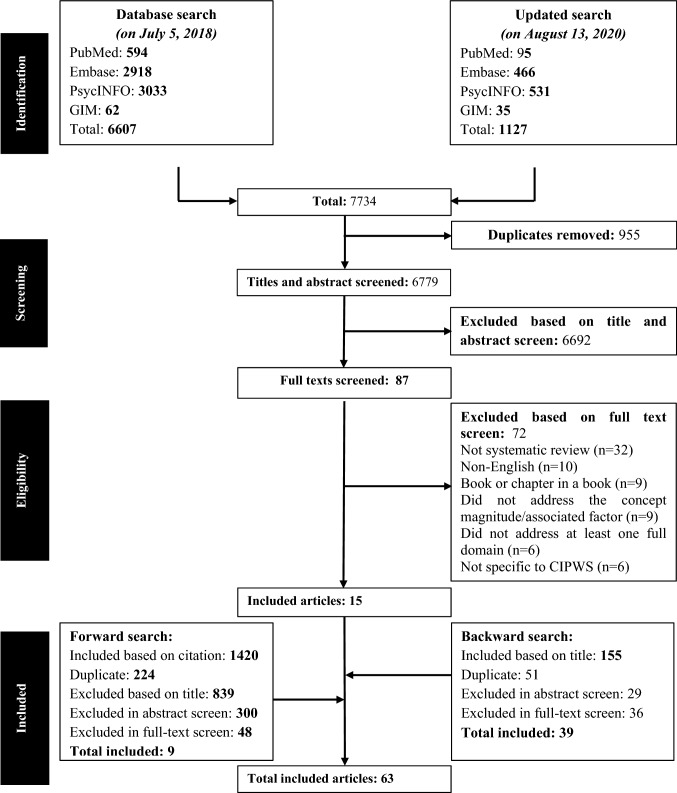
PRISMA (Preferred Reporting Items for Systematic Reviews and Meta-Analysis) flow diagram showing how articles are identified, screened, and included.  Abbreviations: *CIPWS* cognitive impairment in people with schizophrenia

The search yielded a total of 7734 articles (i.e., 6607 articles in the initial search and 1127 on the updated search). Title and abstract screening yielded 87 articles, while full-text screening, and forward and backward searching gave us 63 systematic reviews (Fig. 1). A list of excluded articles after full-text screening with the reason for their exclusion is provided in online resource 3.

All the included systematic reviews evaluated cognitive impairment and associated factors in PWS with no geographical restriction, except one review from China. A diverse group of mental disorders were included in each review. In all the reviews, PWS were either compared within schizophrenia group or with other population groups, such as people with affective disorder or healthy controls. The majority of the reviews searched both PubMed and PsycINFO databases (*n* = 39). The quality of most of the included reviews was moderate (*n* = 38). We identified the following four themes from analysis of the data extracted from the included reviews.

1. Domains of cognition impaired in PWS from specific single cognitive domain reviews (*n* = 15/63)
2. Comparison of cognitive impairment in PWS with different groups from reviews with multiple domains (*n* = 17/63)
3. Course/progress of cognitive impairment in PWS over time (*n* = 6/63)
4. Cognitive impairment in PWS and associated factors (*n* = 25/63)

### Domains of cognition impaired in PWS from specific single cognitive domain reviews

We identified a total of 15 systematic reviews which assessed the magnitude of cognitive impairment in PWS in a specific single cognitive domain. Five assessed memory, four executive function, two processing speed, two verbal fluency, and two social cognition. These reviews were published between 1999 and 2019. The majority of the reviews (*n* = 9/15) searched both PubMed and PsycINFO, and they included from 10 to 124 independent studies (1 review did not report the number of studies included). Ten of the 15 systematic reviews had moderate quality, while the remaining five studies had poor quality. Detailed description of the reviews included are given in (Table 2).

**Table 2:** Characteristics of systematic reviews that assessed domains of cognitive impairment in people with schizophrenia from single domain reviews

Citation	Databases searched	Range of search year	Included studies	Total sample size of studies included	Domain	Concept addressed	Quality assessment
Piskulic et al. (2007)	–PubMed and MEDLINE	–From 1992 to 2005	–33 studies	–1112 PWS and 1241 HC	–SWM	Both^a^	Poor quality
Aleman at al. (1999)	–Psychist and MEDLINE	–From 1975 through July 1998	–70 studies	–60 studies, a total of 3315 in meta-analysis	–Short and long-term memory	Both^a^	Poor quality
Lee and Park (2005)	–PsycINFO and MEDLINE	–From 1980 to September 2004	–124 studies	–Sample size is not reported systematically	–WM	Magnitude, comparison	Moderate quality
Doughty and Done (2009)	–PubMed and PsycINFO	–The start date is NR, search was conducted on October 2007	–91 studies	–Sample size is not reported	–Semantic memory	Magnitude of impairment	Moderate quality
Pelletier et al. (2005)	–PubMed and PsycINFO	–From 1965 to July 2003	–84 studies	–Sample size is not reported systematically	–EM	Both^a^ and comparison	Moderate quality
Johnson-Selfridge and Zalewski (2001)	–MEDLINE, PsycINFO, PsychLit	–Until early 1997	–71 studies	–Sample included is Not reported systematically	–EF	Both^a^	Moderate quality
Dibben et al. (2009)	–MEDLINE, PsycINFO, Embase	–Until March 2008	–88 studies	–Sample size are not reported systematically	–EF	Association with EF impairment	Moderate quality
Li (2004)	–MEDLINE	–Until November 2003	–59 studies	–Sample size is not reported	–EF	Error in the WCST (type of EF problem)	Poor quality
Thai et al. (2019)	–PsycINFO and PubMed	–On 1st September 2017	–10 studies	–375 PWS and 541 HC	–EF	Magnitude and comparison of sub-domains of EF	Moderate quality
Bokat and Goldberg (2003)	–MEDLINE	–Until June 1, 2001	–13 studies	–915(PWS = 526, HC = 389)	–VF	Magnitude and comparison	Poor quality
Henry and Crawford (2005)	–Web of science, Psych lit CD-ROM, and science direct	–Between 1981 and 2002	–84 studies	–2947 PWS and 2469 HC	–Semantic and phonetic VF	Magnitude, and comparison	Moderate quality
Knowles et al. (2010)	–MEDLINE and PsycINFO	–From May 2006 to January 2009	–36 studies	–4,135 PWS and 2,292 HC	–PS	Both^a^	Moderate quality
Dickinson et al. (2007)	–MEDLINE and PsycINFO	–From 1990 to April 2006	–37 studies	–1961 PWS and 1444 HC	–PS	Both^a^	Moderate quality
Savla et al. (2013)	–PsycINFO And PubMed	–From 1980 to November, 2011	–112 studies	–3980 PWS and 3570 HC	–SC	Both^a^	Moderate quality
Javed and Charles (2018)	–PubMed	–Past 10 years	–NR	–NR	–SC	Both^a^	Poor quality

*EF* executive function, *HC* healthy control, *NR* not reported, *PS* processing speed, *PWS* people with schizophrenia, *SC* social cognition, *SWM* spatial working memory, *VF* verbal fluency, *WCST* wisconsin card sorting test, *WM* working memory

^a^Both is for magnitude and associated factors

**Fig. 2:**
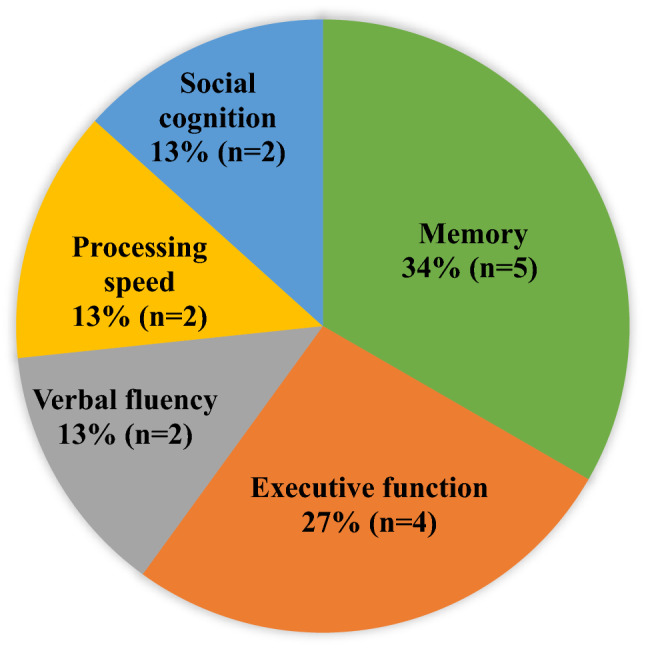
Domain of cognition impaired in people with schizophrenia as extracted from systematic reviews of single domain reviews (*n* = 15)

The most frequently studied domain of cognition is memory; 5 of the 15 systematic reviews [14–18] included were about different kinds of memory impairment in PWS. In all types of memory examined (spatial working memory, short-term and long-term memory, working memory, episodic memory, and semantic memory), PWS were found to have more impairment compared to healthy controls. The second most commonly studied domain was executive function. Four systematic reviews [19–22] included were about the level of impairment in executive function in PWS. All the four reviews reported that PWS had greater impairment in executive function compared to healthy controls. Two reviews [23, 24] were on processing speed. These reviews found that PWS had greater impairment in processing speed compared to controls. The level of impairment of verbal fluency was examined in two systematic reviews [25, 26], which found that PWS were more impaired in verbal fluency compared to healthy controls. Semantic fluency was reported to be more impaired in PWS compared to letter/phonetic fluency. Finally, other two systematic reviews [27, 28] included under this sub-section examined social cognition. Both reviews disclosed that in all domains of social cognition PWS performed worse compared to controls (Fig. 2).

### Comparison of cognitive impairment in PWS with different groups from reviews with multiple domains

A total of 17 systematic reviews compared the magnitude of cognitive impairment in PWS with different groups in multiple domains of cognition. Of these, five reviews compared cognitive impairment in PWS and people with bipolar disorder/affective disorders and healthy controls; four compared cognitive impairment in PWS with healthy controls only. Five systematic reviews compared cognitive impairment in first episode/drug-free PWS with healthy controls. One review compared cognitive impairment in first episode schizophrenia patients with first episode bipolar patients and healthy controls. Two reviews compared cognitive domains to one another. Except for one which was rated as a good review, all the other reviews were of moderate quality (*n* = 12/17) or poor quality (*n* = 4/17) (Table 3).

**Table 3:** Characteristics of systematic reviews that assessed domains of cognitive impairment in people with schizophrenia from multiple domains reviews

Citation	Databases searched	Range of search year	Number of included studies	Total sample size of studies included	Domain	Concept addressed	Quality assessment
Trotta et al. (2015)	–MEDLINE (PubMed), Embase and PsycINFO	–From 1990 to 2013	–28 studies	–For premorbid 1993 PWS, 1270 PWBP, and 772,138 HC –For post-onset 1087 PWS, 811 PWBP and 1400 HC	–General	Comparison with PWBP and association	Moderate quality
Stefanopoulou et al. (2009)	–PsychLit, MEDLINE and Embase	–From January 1980 to December 2005	–53 studies	–2508 PWBD, 1067 PWS, 197 MDD pt and 992 HC	–More than one domain	Magnitude, comparison people with affective d/r and PWS	Moderate quality
Bora et al. (2009)	–PubMed, Scopus, PsycINFO, Embase and MEDLINE	–January 1975 and December 2007	–31 studies	–1979 PWS and 1314 people with affective psychosis or schizoaffective D/r	–More than one domain	Magnitude, comparison with schizoaffective and associated factors	Moderate quality
Krabbendam et al. (2005)	–MEDLINE and PsycLIT	–Between 1985 and October 2004	–31 studies	–Sample size is not reported	–More than one domain	Magnitude, comparison between PWBD and PWS	Moderate quality
Kuswanto et al. (2016)	–NCBI, PubMed/MEDLINE, Scieverse, Scidirect, and Web-of-Science	–From January 2000 to July 2015	–36 studies	–2605 PWS, 1972 PWBD and 2512 HC	–More than one domain	Both^a^, and comparison PWS and people with affective d/r	Poor quality
Schaefer et al. (2013)	–PubMed	–January 2006 through June 2012, an update	–100 studies	–9048 PWS and 8814 HC	–More than one domain	Both^a^ and comparison of PWS and HC	Moderate quality review
Fioravanti et al. (2012)	–PubMed, PsycINFO, PsycArticles	–Up to March 2010	–240 studies	–18,300 PWS	–More than one domain	–Both^a^ and comparison of PWS and HC	Moderate quality review
Fioravanti et al. (2005)	–Psychlit, Psychiatry, and MEDLINE	–Between 1990 and 2003	–113 studies	–4365 PWS and 3429 HC	–More than one domain	Magnitude, comparison of PWS and HC	Moderate quality
Heinrichs and Zakzanis (1998)	–PsycINFO and Medline	–Between 1980 and 1997	–204 studies	–7,420 PWS, and 5,865 HC	–More than one domain	–Both^a^, and comparison, b/n PWS and HC	Poor quality

Citation	Databases searched	Range of search year	Number of included studies	Total sample size of studies included	Domain	Concept addressed	Quality assessment
Mesholam-Gately (2009)	–MEDLINE/PubMed and PsycINFO	–Until October 2007	–47 studies	–2,204 FEPWS and 2,775 largely age- and gender matched HC	–More than one domain	Both^a^ and comparison of FEPWS with HC	Moderate quality
Rajji et al. (2009)	–29 databases (including Embase, MEDLINE and PsycINFO)	–1980 to 2008	–109 studies	–5010 PWS Number of the HC are not systematically reported	–More than one domain	Magnitude, comparison Youth onset schizophrenia	Moderate quality
Fatouros-Bergman et al. (2014)	–PubMed and PsycINFO	–Until 1st of November 2012	–23 studies	–1106 PWS and 1385 HC	–More than one domain	Magnitude, comparison between PWS and HC	Moderate quality
Aas et al. (2014)	–MEDLINE (PubMed) and PsycINFO	–Up to and including January 2013	–24 Studies	–Not reported	–More than one domain	Magnitude, comparison FES and HC	Poor quality
Bora and Pantelis (2015)	–PubMed, PsycINFO, ProQuest, and Scopus	–From January 1990 to July 2014	–22 studies	–533 FEB pt with 1417 HC and 605 FEB pt and 822 FES pt	–More than one domain	Comparison b/n FEB pt and FES pt and association	Moderate quality
Zhang et al. (2019)	–PubMed, Embase, PsycINFO, Cochrane Library, CNKI, WANFANG DATA, WEIPU Journal Net (VIP) and SinoMed	–Up to 13 March 2019	–56 studies only from Chinese	–3167 FES and 3017 HC	–More than one domain	Both^a^, and comparison FES and HC	Moderate quality
Thibaudeau et al. (2019)	–PubMed, PsycINFO, Embase, Proquest, SciVerse, ScienceDirect and Cochrane Library	–1980 and December 19th 2018 (including Epub)	–91 studies	–5462 PWS	–More than one domain	–Relationship b/n ToM and NC and factors affecting the association Magnitude b/n PWS and HC and Comparisons across domains	Good quality
Pickup (2008)	–PsycINFO and MEDLINE	–In May 2007	–17 studies	–Ranges between 16 and 128	–More than one domain	–Relationship b/n ToM and EF –Magnitude b/n PWS and HC and Comparisons across domains	Poor quality

*D/r* disorder, *HC* healthy controls, *NCBI* national centre of bio-technology information, *PWBD* people with bipolar disorder, *PWS* people with schizophrenia, *CNKI* China national knowledge infrastructure, *EF* executive function, *FEBP* first episode bipolar, *FEPWS* first episode people with schizophrenia, *FES* first episode psychosis, *HC* healthy controls, *NC* neuro cognition, *Pt* patients, *PWS* people with schizophrenia, *SinoMed* sino biomedicine service system, *ToM *theory of mind

^a^For both magnitude and associated factors

From the reviews that compared cognitive impairment in PWS and people with bipolar disorder or other affective disorders, we found that PWS had significantly higher cognitive impairment compared to both people with bipolar disorder, or other affective disorders, and healthy controls, whereas people with bipolar disorder had intermediate cognitive impairment compared with PWS and healthy controls [29–33]. Regarding impairment in specific domains, one review [31], reported that greater impairment was observed in verbal fluency, whereas no difference was reported in the domains of delayed verbal memory, and fine motor skill between PWS and people with bipolar disorder. Similarly, another review [30] found that there was no significant group difference in the domains of verbal memory, attention (digit span), and spatial working memory between PWS and people with bipolar disorder.

Compared to healthy controls, PWS scored significantly lower across all cognitive domains [3, 34–36]. Looking at specific domains, greater impairment was found in the domains of processing speed and memory, whereas lower impairment was reported in domains of language and Intelligence Quotient (IQ) [34, 36].

We found that people with first episode schizophrenia were more impaired compared to healthy controls in multiple domains [37–40]. In the domain of verbal memory, greater impairment was reported in two reviews [37, 40]. Compared to people with first episode bipolar disorder cases and healthy controls, people with first episode schizophrenia were found to have worse performance while, people with bipolar disorder were reported to have cognitive impairment worse than healthy controls [41]. Similarly, in one systematic review of studies on cognitive impairment among treatment naive PWS [4], it was reported that PWS performed worse compared to healthy controls in all the domains considered, the three domains with greater impairment were verbal memory, processing speed, and working memory. These systematic reviews confirmed that cognitive impairment in PWS occurred in the absence of medication (i.e., antipsychotics).

Finally, two reviews focused on the relationship among the different cognitive domains. One of the two reviews [42] examined the relationship between theory of mind and neurocognitive domains. This review reported moderate association between theory of mind and each neurocognitive domain (Zrs 0.27–0.43), with no significant difference among domains in the neurocognitive domain. In the same review, stronger association was reported between theory of mind and executive function and abstraction domains. While the second review [43] that compared theory of mind impairment with executive function impairment reported that PWS had greater impairment in both domains and theory of mind continued to predict schizophrenia (rather than being a control participant) once executive function was controlled for. This review supported the hypothesis that theory of mind and executive function impairments in PWS are independent of one another.

### Course/ progress of cognitive impairment in PWS over time

Six of the systematic reviews we identified were about the course of cognitive impairment in PWS [44–49]. Methodologically, two reviews were rated as poor and the other four were rated as moderate quality (Table 4).

**Table 4:** Characteristics of systematic reviews that assessed the course/progress of cognitive impairment in people with schizophrenia

Citation	Databases searched	Range of search year	Number of included studies	Sample size of studies included	Domain addressed	Concept addressed	Quality assessment
Shah et al. (2012)	–PubMed	–Between 1960 and 2009	–23 studies	–Sample size systematically NR	–More than one domain	Course of CIPWS	Poor quality
Irani at al. (2010)	–MEDLINE	–From inception to November 30, 2007 and February 16, 2008,	–43 studies	2110 PWS, 1738 HC form cross-sectional studies and –954 PWS from longitudinal studies	–More than one domain	Course and factors associated	Moderate quality
Bozikas and Andreou (2011)	–PubMed	–Until 15 January 2010	–26 studies	–Sample size is not reported systematically	–More than one domain	Progress and associated factors	Moderate quality
Szöke et al. (2008)	–MEDLINE	–From 1978 to 2006	–53 studies	–2476 PWS and 324 HC	–General	Course and associated factors	Poor quality
Woodberry (2008)	–PubMed	–Until March 1, 2007	–18 studies	–Sample size systematically NR	–IQ	Magnitude, and course	Moderate quality
Khandaker et al. (2011)	–MEDLINE–PubMed, PsycINFO and Embase	–From January 1984 until February 2011	–14 studies	–4396 cases and over 745,000 HC	–IQ	Comparison and course	Moderate quality

*CIPWS* cognitive impairment in people with schizophrenia, *HC* healthy controls, *NR* not reported, IQ intelligence quotient, *PWS* people with schizophrenia

Only one review [44] reported cognitive function decline over time. Even this review reported mixed results with slightly more studies pointing towards decline with a 1 to 1.2 ratio of no decline vs decline. This review was rated as poor quality with our criteria. To the contrary, two moderate-quality reviews [45, 46] reported no decline in cognitive function of PWS from follow-up studies. Most of the included studies in this review showed no difference between those treated with typical and atypical antipsychotics; among various atypical antipsychotics, and between medicated and un-medicated participants in terms of cognitive impairment [46]. One systematic review [47], which is a poor-quality review, reported improvement in cognitive function over time.

Two reviews [48, 49] examined the course of IQ in PWS, and both reviews found that PWS had lower performance in IQ test compared to controls. In both reviews, mixed results (i.e., both decline over time and no decline) were reported, where no decline over time was reported in better quality studies.

### Cognitive impairment in PWS and associated factors

**Fig. 3:**
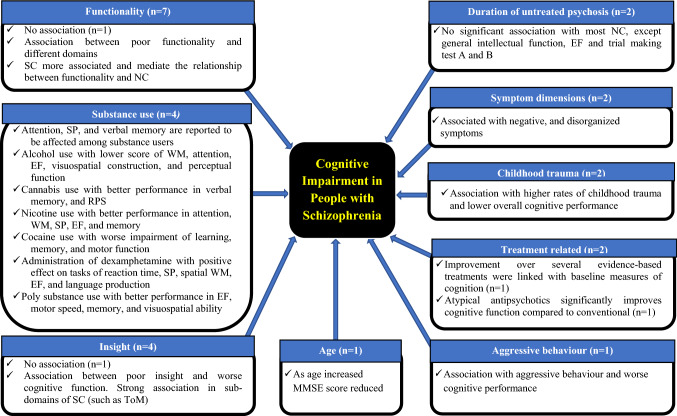
Pictorial presentation of factors associated with cognitive impairment in people with schizophrenia from reviews included (*n* = 25). Abbreviations: *CIPWS* cognitive impairment in people with schizophrenia, *EF* executive function, *MMSE* mini mental state examination, *NC* neuro cognition, *RPS* reasoning and problem solving, *SC* social cognition, *SP* speed of processing, *ToM* theory of mind, *WM* working memory

Twenty-five reviews focused on cognitive impairment in PWS and associated factors such as functionality, symptom dimensions, substance use, age of patients, insight into their problem, childhood trauma, duration of untreated psychosis, treatment, and aggressive behaviour. Seven of the 25 reviews examined the relationship between cognitive impairment and functionality, while four of them evaluated the relationship between cognitive impairment and substance use. Other four reviews examined the relationship between cognitive impairment and insight and two others each examined the relationship between cognitive impairment and symptom dimensions, duration of untreated psychosis, treatment, and childhood trauma. One review each examined the relationship between cognitive impairment and age, and aggressive behaviour (Fig. 3). Twelve of the 25 systematic reviews were moderate quality, while two were high quality (Table 5).

**Table 5:** Characteristics of systematic reviews that assessed the association of cognitive impairment in people with schizophrenia with different factors

Citation	Databases searched and	Range of search year	Number of Included studies	Sample size of studies included	Domain addressed	Concept addressed	Quality assessment
Allott et al. (2011)	–PsycINFO and MEDLINE	–Until 31st July 2010	–22 studies	–Sample size systematically NR	–More than one domain	Association with functionality	Good quality
Christensen (2007)	–PubMed (1966), PsycINFO (1967), and Embase (1974)	–Up to 2004	–21 studies	–Range from 30 to 118 patients: and media*n* = 75	–General	Association with functionality	Moderate quality
Fett et al. (2011)	–MEDLINE and PsycINFO	–January 1977 to August 2009	–52 studies	–2692 people with non-affective psychosis	–More than one domain	Association with functionality	Moderate quality
Couture et al. (2006)	–PsycINFO and MEDLINE/ PubMed	–Range of search year is NR	–NR	–Sample size included are systematically NR	–SC	Association of SC with functionality	Poor quality
Rajji et al. (2014)	–MEDLINE	–1999 to June 20, 2012	–NR	–NR	–More than one domain	Association with functionality from Long-studies	Poor quality
Halverson et al. (2019)	–PsycINFO, PubMed, and ProQuest PsyArXiv	–Update of Fett et al. (2011) to July 2017	–166 studies	–12,868 PWS; 518 for correlation study	–More than one domain	Association with functionality	Good quality
Schmidt et al. (2011)	–MEDLINE and PsycINFO	–January 2004 and December 2010	–15 studies	–Ranges from 26 to 178	–More than one domain	Association with functionality	Poor quality
Donoghue and Doody (2012)	–PsycINFO, MEDLINE, Zetoc Embase, CINAHL, Web of Knowledge	–1980 and up until October 2010	–21 studies	–Comorbid diagnosis of a SUD was 763, and the totals with no comorbid diagnosis was 770	–More than one domain	Association with substance	Moderate quality
Yüce et al (2010)	–PubMed, PsycINFO, Scopus	–Between 1987 and March 2010	–10 studies	–572 PWS (with and without cannabis use)	–More than one domain	Cannabis as an associated variable	Moderate quality
Potvin et al. (2008)	–PubMed and PsycINFO	–Until 31st August 2006	–23 studies	–1807 PWS with/without SUD, 789 PWS + SUD and 1106 PWS	–More than one domain	Substance as an associated variable	Moderate quality
Coulston et al. (2007)	–MEDLINE and PsycINFO	–NR	–65 studies	–Sample size are not systematically NR	–More than one domain	Substance as an associated variable	Poor quality
Dominguez et al. (2009)	–MEDLINE and PsychINFO	–January 1977 to April 2008	–58 studies	–5,009 people with non-affective psychosis	–More than one domain	Association with sx dimensions	Moderate quality
Nieuwenstein et al. (2001)	–Psychlit and Medline	–1980 to December 1999	–For WCST 16, and for CPT 6 studies	–Sample size are not reported systematically	–EF and At/vigilance	Association with sx dimensions	Poor quality

Citation	Databases searched	Range of search year	Number of Included studies	Sample size of studies included	Domain addressed	Concept addressed	Quality assessment
Maltais et al. (2015)	–PubMed and PsycINFO	–Between 1975 and 2012	–56 studies	–5,588 PWS	–General	Association with age	Poor quality
Kurtz (2011)	–PsycINFO (2010), MEDLINE (2008)	–1980 up to 2008/2010	–20 studies	–Sample size is not systematically reported	–More than one domain	Response for treatment	Poor quality
Keefe et al. (1999)	–MEDLINE and Psychology Abstract	–Between 1990 and April 1998	–15 studies	–Sample size are not reported systematically	–More than one domain	Comparison b/n typical and atypical antipsychotics	Moderate quality
Subotnik et al. (2020)	–PsychINFO, PubMed, and Google Scholar	–January 1, 1993 to June 30, 2018	–123 studies	–14,932 PWS	–More than one domain	Association with insight	Poor quality
Nair at al. (2014)	–PubMed and Web of Science	–For insight study from 2004 until September 2012	–72 studies for insight study, 34 studies in PWS	–5429 patients for insight study, 1821 PWS	–More than one domain	Association with insight in psychotic disorder	Moderate quality
Agrawal at al. (2003)	–PubMed and Web of Science	–1980 and April 2004	–35 studies	–2354 people with psychosis	–More than one domain	Association with insight	Moderate quality
Cooke et al. (2005)	–PsycINFO (from1967) and MEDLINE (from 1966)	–Up to December 2004	–45 studies	–Range is between 21 and 535	–More than one domain	Association with insight	Poor quality
Reinharth et al. (2014)	–PsycINFO and MEDLINE	–Up to April 2013	–29 studies	–4764 PWS	–More than one domain	Association with Aggressive behaviour	Moderate quality
Vargas et al. (2019)	–PubMed, Ovid MEDLINE, and Web of Science	–On July 28, 2018	–23 studies	–3315 people with Psychotic	–More than one domain	Association with childhood trauma	Moderate quality
Dauvermann and Donohoe (2019)	–PubMed and PsycINFO	–Between 1993 and December 2017	–21 studies	–Range b/n 28 and 659	–More than one domain	Association with childhood trauma	Poor quality
Allott et al. (2018)	–PubMed and Embase	–Inception to June 2015	–43 studies	–4647 FEP patients	–More than one domain	Association with DUP	Poor quality
Bora et al. (2018)	–PubMed and Scopus	–January 1980 to February 2017	–27 studies	–3127 FEP	–More than one domain	Association with DUP	Moderate quality

*CPT* continuous performance test, *Long-studies* longitudinal studies, *NR* not reported, *PWS* people with schizophrenia, *SC* social cognition, *SUD* substance used disorder, *Sx* symptom; *WCST* wisconsin card sorting test, *At* attention, *DUP* duration of untreated psychosis, *EF* executive function, *FEP* first Episode Psychosis, *PWS* people with schizophrenia

As shown in (Fig. 3), the relationship between cognitive impairment in PWS and functionality was examined in seven systematic reviews [50–56]. One good-quality review [50] concluded that there was no association between cognition and functional outcome. However, the other six reviews [51–56] found some association between different domains of cognition and different aspects of functionality. Three of these reviews were poor quality, two moderate quality, and one good-quality review (Fig. 3 and Table 5).

Four reviews [57–60] examined the relationship between substance use and cognitive impairment in PWS. These reviews reported inconsistent findings. The effect of substance use in cognitive impairment in PWS differed from substance to substance, and from domain to domain (Fig. 3 and Table 5).

Four reviews that examined the association between cognitive impairment in PWS and insight [61–64] reported mixed findings. One poor-quality review [62] reported inconsistent results, with more studies reporting no association between insight and neurocognition. While the other three reported a significant relationship between poor insight and worse cognitive performance both in neurocognitive and social cognitive domains [61, 63, 64], with stronger relationship in some sub-domains of social cognition (such as theory of mind) [64] (Fig. 3 and Table 5).

With regards to the relationship between cognitive impairment and symptom dimensions, two reviews [65, 66] were included in the umbrella review. These reviews found that cognitive impairment was associated with negative symptoms and disorganized symptoms compared to positive symptoms and depressive symptoms (Fig. 3 and Table 5).

Two reviews examined the relationship between cognition and duration of untreated psychosis [67, 68]. One of these had poor quality and the other one moderate quality. Both concluded that there was no significant association between most neurocognition domains and duration of untreated psychosis, with the exception of general intellectual function, executive function, and trial making test A and B (Fig. 3 and Table 5).

Two reviews examined the relationship between childhood trauma and cognitive impairment in PWS [69, 70]. Both reviews reported significant association between higher rates of childhood trauma and reduced overall cognitive performance; one of these reviews was poor quality and the other one was moderate quality (Fig. 3 and Table 5).

Two reviews [71, 72] were about the relationship between cognitive impairment and treatment. One of these reviews [71] was about the effect of cognitive impairment on response to different forms of treatment. In this review, it was shown that improvement over several evidence-based treatments were linked with baseline measures of cognitive function. The other review was comparing the effect of typical and atypical antipsychotics in treating cognitive impairment in PWS [72]. This review reported that atypical antipsychotics significantly improved cognitive function compared to conventional antipsychotics. This review also concluded that the studies that were included had many methodological limitations that one needs to consider in interpreting the findings (Fig. 3 and Table 5).

One of the reviews we included [73] dealt with the association between cognitive impairment and age. The study concluded that age had direct effect on the score of mini-mental state examination (MMSE); for every four years increase in age of PWS, there was one MMSE point reduction (five times higher than the report in the general population). PWS living in institutions were more impaired compared to those who were living in the community. This was of a poor-quality review (Fig. 3 and Table 5).

The other review included under this section examined the association between cognitive impairment in PWS and aggressive behaviour [74]. This moderate-quality review concluded that cognitive impairment in PWS exerted a significant risk for aggression (Fig. 3 and Table 5).

## Discussion

Multiple studies have been published on the magnitude of cognitive impairment in PWS and associated factors. We were able to identify 63 independent reviews, and the majority addressed several neurocognitive domains. Most of the reviews used PubMed and PsycINFO databases to search their included studies. Methodologically, the majority of the studies were of moderate quality.

Of the 63 reviews we identified, some (*n* = 24) were included in a previous umbrella review of the magnitude of cognitive dysfunction in PWS and people with bipolar disorder [9]. The difference between our review and this previous umbrella review is that, the former only included meta-analysis studies while our review included both meta-analysis and systematic reviews. This previous review was not specific to cognitive impairment in PWS rather it was an umbrella review of cognitive impairment in PWS and people with bipolar disorder. It also focused more on comparing the domains of cognitive impairment in PWS and people with bipolar disorder. The previous review only included meta-analysis of neurocognitive domains, ignoring social cognitive domains, which is considered to be an important predictor of functionality. Finally, the previous review was not comprehensive (searched PubMed only) and the last date of search for the previous umbrella review was on August 10th 2015 (5 years older than ours). These facts make our review more comprehensive compared to it.

Even though the area is extensively researched, none of the included reviews reported the problem from low and middle-income countries (LMICs), separately. Since there are reports which show that cognition can be affected by nutritional status [75], we expect different results in high-income countries compared to that of LMICs. This calls for a separate analysis of the magnitude of cognitive impairment and associated factors based on income level of countries. One of the reasons for the lack of separate analysis may be scarcity of studies from LMICs. Therefore, conducting studies focusing on the magnitude, associated factors, and other interventional studies might be worth considering.

In this umbrella review, we found that PWS have more severe cognitive impairment compared to both healthy controls, people with bipolar disorder, and people with other affective disorders, particularly in the domains of processing speed, verbal memory, and working memory. We also found that the magnitude of cognitive impairment is more or less the same in PWS who were drug free and first episode psychosis, and those who had taken pharmacological treatment. Even though the results were mixed, most of the reviews we included point toward no decline of cognitive impairment in PWS over time. These findings support the hypothesis that cognitive impairment is one of the core symptoms of schizophrenia even at the initial phase of the illness.

This review of reviews showed that cognitive impairment in PWS has significant association with a number of factors, including functionality, substance use, insight, symptom dimensions, history of childhood trauma, older age, and aggressive behaviour. Hence, we recommend future researchers to consider these factors in conducting a study related to factors associated with cognitive impairment in PWS.

This umbrella review is particularly useful for researchers, clinicians, and experts in the area. The implication of this review for research is that it would help researchers in identifying a summary of factors that are potentially associated with cognitive impairment in PWS when planning studies. This study will also have implication for interventional studies that future researchers may design focusing on domains reported to be impaired. Another potential implication of this review is for experts in the area. Experts can refer this review in designing policy and strategies related to cognition in PWS. Clinicians can refer to this review to have a comprehensive understanding of domains of cognition affected and factors associated with cognitive impairment in PWS.

This review has a number of strengths in that we followed PRISMA guideline in conducting and reporting the review. Our search can be considered more comprehensive compared to the one conducted before since we included four databases without restriction on the date of publication, and we also conducted forward and backward searching. Furthermore, we assessed the quality of each of the included reviews, and whenever possible results were presented separately considering the methodological quality of the reviews included.

However, our review is not free from limitations. First, the broad scope of the review made the data analysis difficult, particularly to conduct meta-analysis. The broad scope of the review allowed a wide variety of study outcomes to be included and hence made the review hard to follow. Second, non-English studies were excluded, which might limit the generalizability of the findings. Finally, the grey literature was not searched; however, we used Google Scholar for our forward and backward searching.

## Conclusions

This umbrella review highlights cognition as an important factor contributing to functionality and insight in PWS. Considering the presence of 63 different reviews and no review looked for cognitive difference across the economic classification, we encourage reviews that compare cognitive impairment in PWS at different income settings. From this umbrella review, one can conclude that cognitive impairment is a core symptom in PWS and different factors such as functionality, insight, history of childhood trauma, age, and aggressive behavior have significant association with cognitive impairment in this population group.

## Supplementary Information

Below is the link to the electronic supplementary material.

Supplementary file1 (DOCX 14 KB)

Supplementary file2 (XLSX 10 KB)

Supplementary file3 (DOCX 170 KB)

## Data Availability

All the data used are made available in the manuscript and supplementary materials.

## Abbreviations

AMSTAR: A measurement tool to assess methodological quality of systematic review
DSM: Diagnostic and statistical manual of mental disorders
ICD: International classification of diseases
IQ: Intelligence quotient
LMIC: Low- and middle-income countries
MATRICS: Measurement and treatment research to improve cognition in schizophrenia initiative
MMSE: Mini-mental state examination
PANSS: Positive and negative syndrome scale
PRISMA: Preferred reporting items for systematic reviews and meta-analyses
PWS: People with schizophrenia
