# Supplementary material for: Cognitive impairment in people with schizophrenia: an umbrella review
Source: Eur Arch Psychiatry Clin Neurosci. 2022 May 28;272(7):1139–55. doi: 10.1007/s00406-022-01416-6 (PMC9508017; doi:10.1007/s00406-022-01416-6)
Supplement: Supplementary file 1 — Supplementary file1 (DOCX 14 KB) [file 406_2022_1416_MOESM1_ESM.docx]

Online resource 1: Search strategy for an umbrella review of cognitive impairment in people with schizophrenia

| S.N. | Big terms | Free terms | Mesh terms | Emtree | PyschINFO  Terms |
| --- | --- | --- | --- | --- | --- |
| 1 | **Big term 1:**  “Cognitive function” | Cognit* OR Neurocognit* OR Neuropsycholog* | "Cognition Disorders"[Majr] | exp cognition/ or exp cognitive defect or exp neuropsychology/ | exp cognition/ or exp Cognitive Ability/ or exp Cognitive Impairment/  exp neuropsychology/ |
| 2 | **Big term 2:**  Schizophrenia | Schizophren* OR Psychosis OR Psychotic OR “Severe mental disorder” OR ScZ OR SMD | "Schizophrenia Spectrum and Other Psychotic Disorders"[Majr] | exp schizophrenia/ or exp psychosis/ | exp schizophrenia/ or exp PSYCHOSIS/ |
| 3 | **Big term 3:**  Prevalence  OR  Determinants | Prevalence OR Incidence OR Magnitude OR Proportion OR “Associated factor” OR “Risk factor” OR Predictor OR “Factor affecting” OR “Factor associated” OR Determinant | "Prevalence"[Majr] OR "Incidence"[Majr] OR "Risk Factors"[Majr] | exp prevalence/  exp incidence/  exp risk factor/ | exp risk factor/ |
| 3 | **Objective 4 and 5** | 1 AND 2 AND 3 |  |  |  |
