# Supplementary material for: Cognitive impairment in people with schizophrenia: an umbrella review
Source: Eur Arch Psychiatry Clin Neurosci. 2022 May 28;272(7):1139–55. doi: 10.1007/s00406-022-01416-6 (PMC9508017; doi:10.1007/s00406-022-01416-6)
Supplement: Supplementary file 3 — Supplementary file3 (DOCX 170 KB) [file 406_2022_1416_MOESM3_ESM.docx]

# List of excluded articles

Online resource 3: List of excluded articles with reason of exclusion

| S.N. | **Citation (Author and year)** | Title | **Reason for excluded articles** |
| --- | --- | --- | --- |
| 1 | (Bowie and Harvey, 2006) | Cognitive deficits and functional outcome in schizophrenia | Not a systematic review; it is a kind of scoping review |
| 2 | (Spencer et al., 2017) | Diversity or disarray? A systematic review of decision-making capacity for treatment and research in schizophrenia and other non-affective psychoses | It is a systematic review but doesn’t address the concept of interest (it is about decision making ability of PWS for research and treatment) |
| 3 | (Sprong et al., 2007) | Theory of mind in schizophrenia | It is a systematic review, but doesn’t address one full domain of CIPWS (it is about ToM, a sub-domain of SC) |
| 4 | (Kaladjian et al., 2012) | Schizophrenia and/or bipolar disorder: The neurocognitive endophenotypes | Language (French) |
| 5 | (Kurtz and Marcopulos, 2012) | Cognition in schizophrenia | Not a systematic review (it is chapter in a book) |
| 6 | (Billeke and Aboitiz, 2013) | Social cognition in schizophrenia: from social stimuli processing to social engagement | Not a systematic review (it is a kind of scoping review, with no search term and no database searched) |
| 7 | (Moritz and Woodward, 2006) | Metacognitive control over false memories: A key determinant of delusional thinking | Not a systematic review (it is a kind of experts’ review, with no search term and no database searched) |
| 8 | (Harvey, 2005) | Cognition and Function in Older Patients With Schizophrenia | Not a systematic review (it is chapter in a book) |
| 9 | (Landgraf et al., 2012) | Cognitive identity in schizophrenia: vision, space, and body perception from prodrome to syndrome | Not a systematic review (it is a kind of overview, with no search term and no database searched) |
| 10 | (Green et al., 2000) | Neurocognitive Deficits and Functional Outcome in Schizophrenia: Are We Measuring the "Right Stuff"? | Not systematic review, it is a literature review (not systematically structured) of effect of cognition on functionality. |
| 11 | (Harvey et al., 2013) | Hospitalization and Psychosis: Influences on the Course of Cognition and Everyday Functioning in People with Schizophrenia | Not systematic review, it is a literature review (not systematically structured) of relationship of hospitalization and cognition. |
| 12 | (Ventura et al., 2010) | Disorganization and Reality Distortion in Schizophrenia: A Meta-Analysis of the Relationship between Positive Symptoms and  Neurocognitive Deficits | It doesn’t address the disease of inters |
| 13 | (Ventura et al., 2013b) | Neurocognition and symptoms identify links between facial recognition and emotion processing in schizophrenia: Meta-analytic finding | It is a systematic review, but doesn’t address one full domain of CIPWS (it is about facial recognition and emotion processing, two sub-domains of SC) |
| 14 | (Ahmed et al., 2015) | Brain-derived neurotrophic factor (BDNF) and neurocognitive deficits in people with schizophrenia: A meta-analysis | It doesn’t address the disease of inters |
| 15 | (Armando et al., 2012) | COMT implication in cognitive and psychiatric symptoms in chromosome 22q11 microdeletion syndrome: a selective review | It is not a systematic review, rather it is a selective review of the association between cognitive symptoms of schizophrenia and COMT |
| 16 | (Armando et al., 2013) | An overview of correlations between schizophrenia  And 22q11.2 deletion syndrome | It is a systematic review, but it is not majorly about cognitive disorder in PWS schizophrenia, rather it is generally about relationship of schizophrenia and 22q11.2 deletion syndrome. |
| 17 | (Harvey et al., 2006) | Cognition and aging in psychopathology: Focus on schizophrenia and depression | Is not systematic review rather it is an overview of the effect of aging on cognition in PWS and PWD |
| 18 | (Koren and Harvey, 2006) | Closing the Gap Between Cognitive Performance and Real-World Functional Outcome in Schizophrenia: The importance of Metacognition | It is not systematic review rather it is an overview of meta cognition as a moderator between neuro cognition and function outcome |
| 19 | (Kurtz, 2012) | Neurocognition and functional outcome in schizophrenia | It is not a systematic review, rather it is chapter in a book |
| 20 | (Fusar-Poli et al., 2012) | Cognitive Functioning in Prodromal Psychosis: A Meta-analysis | It is a systematic review, but it is not in PWS |
| 21 | (McGurk and Mueser, 2013) | Cognition and work functioning in schizophrenia | It is not a systematic review, rather it is a chapter in a book. |
| 22 | (Nestor et al., 2010) | Distinct Contribution of Working Memory and Social Comprehension Failures in Neuropsychological Impairment in Schizophrenia | It is not a systematic review, rather it is a single study |
| 23 | (Papa and Bersani, 2007) | Face, identity and emotion in schizophrenia: From perception to expression | Language (Italian) |
| 24 | (Piskulic and Addington, 2011) | Social cognition and negative symptoms in psychosis | It is not a systematic review, rather it is a single study |
| 25 | (Rajakumar, 2017) | Prefrontal cortical abnormalities in cognitive deficits of schizophrenia | It is not a systematic review, rather it is a chapter in a book. |
| 26 | (Schnur and Hoffman, 2010) | Nicotinic modulation of attentional deficits in schizophrenia | It is not a systematic review, rather it is a chapter in a book. |
| 27 | (Tyburski et al., 2015) | Neuropsychological Characteristics of Verbal and Non-Verbal Fluency in Schizophrenia Patients | It is not a systematic review, but it is a kind of scoping review about fluency |
| 28 | (Varese and Bentall, 2011) | The metacognitive beliefs account of hallucinatory experiences: A literature review and meta-analysis | It is a systematic review, but it doesn’t address at least one full domain from the main domains identified |
| 29 | (Ventura et al., 2009) | Symptoms as mediators of the relationship between neurocognition and functional outcome in schizophrenia: A meta-analysis | It is doesn’t address the concept of interest |
| 30 | (Ventura et al., 2013a) | Symptom Domains and Neurocognitive Functioning Can Help Differentiate Social Cognitive Processes in Schizophrenia: A Meta-Analysis | It is doesn’t address the concept of interest |
| 31 | (Weinberger et al., 2007) | Neurocognitive Effects of Nicotine and Tobacco in  Individuals with Schizophrenia | Not a systematic review, it is a kind of overview |
| 32 | (Komlosi et al., 2008) | The relationship between cognition and functional outcome in schizophrenia | Language, Hungarian |
| 33 | (Sarolta et al., 2008) | The relationship between cognition and functional outcome in schizophrenia | Language, Hungarian |
| 34 | (Wolf et al., 1991) | The association of tardive dyskinesia with cognitive deficits: A review | Not a systematic review, it is a kind of overview |
| 35 | (Cotter et al., 2018) | Social cognitive dysfunction as a clinical marker: A systematic review of meta-analyses across 30 clinical conditions | It does not address the concept of interest |
| 36 | (Keri and Janka, 2004) | Critical evaluation of cognitive dysfunctions as endophenotypes of schizophrenia | It does not address the concept of interest |
| 37 | (Lungu et al., 2013) | The Incidence and Nature of Cerebellar Findings in Schizophrenia: A Quantitative Review of fMRI Literature | It does not address the concept of interest |
| 38 | (Reichenberg, 2005) | Cognitive impairment as a risk factor for psychosis | It is not a systematic review; it is more of scoping review. |
| 39 | (Song et al., 2015) | Theory of mind in Koreans with schizophrenia: A meta-analysis | It is a systematic review, but doesn’t address one full domain of CIPWS (it is about ToM a sub-domain of SC) |
| 40 | (Bhattacharya, 2015) | Cognitive Function in Schizophrenia: A Review | It is not a systematic review it is an overview about schizophrenia |
| 41 | (Bora, 2015) | Developmental trajectory of cognitive impairment in bipolar disorder: Comparison with schizophrenia | It is not a systematic review it is an overview about cognition in Bp and schizophrenia |
| 42 | (Bora et al., 2009a) | Theory of mind impairment in schizophrenia: Meta-analysis | It is a systematic review, but it does not address one full domain, it is a metanalysis of ToM impairment in PWS |
| 43 | (Frangou, 2013) | Neurocognition in Early-Onset  Schizophrenia | It is not a systematic review it is an overview about cognition in EOS |
| 44 | (Harvey, 2001) | Cognitive impairment in elderly patients with schizophrenia: Age related changes | It is not a systematic review, rather it is an overview of cognition in old age |
| 45 | (Lenka et al., 2017) | Pattern of cognitive impairment in patients with Parkinson's disease and psychosis: A critical review | It is not in PWS, it is about cognitive impairment in PW Parkinson’s disease with psychosis |
| 46 | (Lewis and Glausier, 2016) | Alterations in prefrontal cortical circuitry and cognitive dysfunction in schizophrenia | Not a systematic review, rather it is chapter in a book. |
| 47 | (Loberg and Hugdahl, 2009) | Cannabis use and cognition in schizophrenia | Not a systematic review, rather it is a kind of narrative review |
| 48 | (Ohi et al., 2017) | A Brief Assessment of Intelligence Decline in Schizophrenia As Represented by the Difference between Current and Premorbid Intellectual Quotient | Not a systematic review, rather it is a kind of overview. |
| 49 | (Okruszek and Pilecka, 2017) | Biological motion processing in schizophrenia - Systematic review and meta-analysis | It is a systematic review, but it doesn’t address one full domain of CIPWS, it is about biological motion processing a sub-domain of SC |
| 50 | (Raucher-Chene et al., 2011) | Schizophrenia in the elderly: Clinical, cognitive and social features | Language, French |
| 51 | (Schmi et al., 2011) | Symptoms and Cognition in Geriatric Schizophrenia | Language, German |
| 52 | (Stip, 2006) | Cognition, schizophrenia and the effect of antipsychotic | Language, French |
| 53 | (Vyas et al., 2017) | Theory of Mind in the Early Course of Schizophrenia | It is not a systematic review, rather it is a kind of literature review. In addition, it is about ToM a sub-domain of SC |
| 54 | (Bozikas et al., 2004) | Study of cognitive function in patients with schizophrenia | Language, Greek |
| 55 | (Hahn, 2000) | Cognitive decline in late-life schizophrenia and alzheimer's disease: A longitudinal comparison of performance on the Mini-Mental Status Exam | Not a systematic review, rather it is a dissertation |
| 56 | (Jaaskelainen et al., 1997) | Cognitive deficits in schizophrenia | Language, Finnish |
| 57 | (Janovic et al., 2006) | Cognitive impairment in schizophrenia | Language, Serbo-Croatia |
| 58 | (Jellinger, 2001) | Dementia as a complication of schizophrenia | Not a systematic review, rather it is an abstract in a conference |
| 59 | (Keri and Janka, 2003) | Cognitive dysfunctions in schizophrenia: Are they endophenotypes? | Language, Hungarian |
| 60 | (Krivoy et al., 2012) | The cognitive deficit in schizophrenia | Language, Hebrew |
| 61 | (Ringdahl, 2015) | Impaired theory of mind in psychotic and affective disorders | Not a systematic review, rather it is a dissertation |
| **During backward search** | | | |
| 1 | (Green et al., 2011) | Social Cognition in Schizophrenia, Part 1: Performance Across Phase of Illness | Not a systematic review, it is a single paper that address look for difference in SC across three clinical groups |
| 2 | (Mandelman and Grigorenko, 2012) | BDNF Val66Met and cognition: all, none, or some? A meta-analysis of the genetic association | Is a systematic review but not specific to schizophrenia |
| 3 | (Rund, 1998) | A Review of Longitudinal Studies of Cognitive Functions in Schizophrenia Patients | It is a review but doesn’t use at least one database |
| 4 | (Sevy et al., 2007) | Iowa Gambling Task in Schizophrenia: A Review and New Data in Patients with Schizophrenia and Co-Occurring Cannabis Use Disorders | It is not a review; it is a single study |
| 5 | (Van Winkel et al., 2008) | Psychosocial Stress and Psychosis. A Review of the Neurobiological Mechanisms and the Evidence for Gene-Stress Interaction | It is not specific to CIPWS, |
| 6 | (Ahmed and Bhat, 2014) | Psychopharmacological Treatment of Neurocognitive Deficits in People with Schizophrenia: A Review of Old and New Targets | It is not a systematic review; it is a general overview of cognition in schizophrenia |
| 7 | (Ahmed et al., 2013) | Bipolar Disorders: Symptoms, Management, and Risk Factors | Not systematic review, it is chapter in a book |
| 8 | (Allen et al., 2007) | Factor analytic support for social cognition as a separable cognitive domain in schizophrenia | Not a systematic review, it is a single study |
| 9 | (Antonova et al., 2004) | The relationship between brain structure and neurocognition in schizophrenia: a selective review | Does not address the concept of interest |
| 10 | (Aylward et al., 1984) | Intelligence in Schizophrenia: Meta-Analysis of the Research | Not systematic review, it is a kind of overview of IQ in schizophrenia |
| 11 | (Burns and Patrick, 2007) | Social functioning as an outcome measure in schizophrenia studies | It is a systematic review, but it does not address the concept of inters, it is about measures used to assess social cognition |
| 12 | (Cantor-Graae et al., 2001) | Substance abuse in schizophrenia: a review of the literature and a study of correlates in Sweden | It is not a systematic review; in addition, it is not about cognition in schizophrenia |
| 13 | (Daban et al., 2002) | Correlation between clinical syndromes and neuropsychological tasks in unmedicated patients with recent onset schizophrenia | It is not a systematic review; it is a single study |
| 14 | (Dickson et al., 2012) | Meta-analyses of cognitive and motor function in youth aged 16 years and younger who subsequently develop schizophrenia | It is a systematic review, but is not among PWS rather it is among youngest who with high risk of developing schizophrenia |
| 15 | (Elie et al., 2010) | Cognitive effects of antipsychotic dosage and polypharmacy: a study with the BACS in patients with schizophrenia and schizoaffective disorder | It is not a systematic review, rather it is a cross-sectional study |
| 16 | (Green, 2016) | Impact of Cognitive and Social Cognitive Impairment on Functional Outcomes in Patients With Schizophrenia | Not a systematic review; it is an overview of the impact of cognition on functionality |
| 17 | (Green et al., 2015) | Social cognition in schizophrenia | Not a systematic review; it is an overview about schizophrenia, focusing on social cognition. |
| 18 | (Green et al., 2004) | Longitudinal studies of cognition and functional outcome in schizophrenia: implications for MATRICS | Not a systematic review; it lacks a specific search term and database to be searched it is a kind of overview |
| 19 | (Grube et al., 1998) | Meta-analysis of symptom factors in schizophrenia | It is a systematic review but, it is not about cognitive impairment, it is general about symptoms of schizophrenia |
| 20 | (Harvey and Keefe, 2001) | Studies of Cognitive Change in Patients With Schizophrenia Following Novel Antipsychotic Treatment | Not a systematic review; it is an overview with no search term |
| 21 | (Hemsley, 1993) | A simple (or simplistic?) cognitive model for schizophrenia. | It is not a systematic review; it lacks to have a search term and database searched |
| 22 | (Hill et al., 2004) | Pre-treatment and longitudinal studies of neuropsychological deficits in antipsychotic-naive patients with schizophrenia | It is not a systematic review; it is a single study |
| 23 | (Hoff and Kremen, 2003) | Neuropsychology in schizophrenia: an update | It is not a systematic review, it is an overview about CIPWS with focus on memory, attention, and executive function |
| 24 | (Hori et al., 2006) | Antipsychotic medication and cognitive function in schizophrenia | It is not a systematic review; it is a single study |
| 25 | (Kambeitz et al., 2012) | Effect of BDNF val66met polymorphism on declarative memory and its neural substrate: A meta-analysis | It is a systematic review, but it is not about CIPWS, it is general about the declarative memory in general |
| 26 | (Kremen et al., 1994) | Neuropsychological Risk Indicators for Schizophrenia: A Review of Family Studies | It is not a in disease of inters, it is on families of PWS |
| 27 | (Kurtz, 2005) | Neurocognitive impairment across the lifespan in schizophrenia: an update | It is not a systematic review; it is a critical review on which it has search term and searched in a database but not presented systematically |
| 28 | (Large et al., 2011) | Cannabis Use and Earlier Onset of Psychosis | It is a systematic review; but it doesn’t address the concept of interest |
| 29 | (Laws, 1999) | A Meta-analytic Review of Wisconsin Card Sort Studies in Schizophrenia: General Intellectual Deficit in Disguise? | It is not a systematic review; it is a kind of overview |
| 30 | (Lindenmayer et al., 1995) | Five factor model of schizophrenia: replication across samples | It is not about the disease of the interest |
| 31 | (Malla et al., 2002) | Symptoms, cognition, treatment adherence and functional outcome in first-episode psychosis | Not a systematic review; it is a single study |
| 32 | (Niizato et al., 2001) | Cognitive decline in schizophrenics with alzheimer's Disease: a mini review of neuropsychological and Neuropathological studies | It is not a systematic review; rather it is an overview of the presence of AD in PWS |
| 33 | (Roy and DeVriendt, 1994) | Positive and negative symptoms in schizophrenia: a current overview | Language; French |
| 34 | (Rund, 2009) | Is schizophrenia a neurodegenerative disorder? | It is not a systematic review; it is an overview of studies |
| 35 | (Schimmelmann et al., 2007) | Pre-treatment, baseline, and outcome differences between early-onset and adult-onset psychosis in an epidemiological cohort of 636 first-episode patients | It is not a systematic review; it is a single study |
| 36 | (Schürhoff et al., 2003) | Apolipoprotein E in Schizophrenia: A French Association Study and Meta-Analysis | It is not specific to CIPWS |
| 37 | (Schwartz et al., 2001) | Neurophysiologic mechanisms of attention: a selective review of early information processing in schizophrenics | It is not a systematic review, rather it is a selective review |
| 38 | (Scott et al., 2007) | Neurocognitive Effects of Methamphetamine: A Critical Review and Meta-analysis | It is not a systematic review; in addition, it is not in a disease of interest |
| 39 | (Strauss et al., 2006) | A Compendium of Neuropsychological Tests: Administration, Norms, and Commentary | It is not a systematic review; rather it is a book |
| 40 | (Stuart et al., 1999) | The three-syndrome model of schizophrenia: Meta-analysis of an artefact | It is not about CIPWS |
| 41 | (Thompson et al., 2011) | Social cognition deficits and the ‘ultra-high risk’ for psychosis population: a review of literature | It is a systematic review, but it is not in a disease of inters |
| 42 | (Villalta-Gil et al., 2006) | Neurocognitive performance and negative symptoms: Are they equal  in explaining disability in schizophrenia outpatients? | It is not a systematic review; rather it is a single study |

| **Update on 13^th^ August, 2020** | | | |
| --- | --- | --- | --- |
| 1 | (Acuna-Vargas and Thibaut, 2019) | Cognition in psychiatry | Is not a systematic review, rather it is an editorial |
| 2 | (Calafato and Bramon, 2019) | The interplay between genetics, cognition and schizophrenia | Is not a systematic review, rather it is kind of commentary |
| 3 | (Hamilton, 2019) | The Utility of Neurocognitive Profiles in Diagnosing Comorbid Schizophrenia and Intellectual | Is not a systematic review, rather it is dissertation |
| 4 | (Kalin, 2020) | Psychotic Experiences, Cognitive Decline, and Genetic Vulnerabilities in Relation to Developing Psychotic Disorders | Is not a systematic review, rather it is Editorials note |
| 5 | (Kimoto et al., 2019) | Neurobiology and treatment of social cognition in schizophrenia: Bridging the bed-bench gap | Is not a systematic review, rather it is kind of overview |
| 6 | (Luther et al., 2018) | A meta-analytic review of self-reported, clinician-rated, and performance-based motivation measures in schizophrenia: Are we measuring the same “stuff” | It is a systematic review but doesn’t address the concept of interest (it is about measures of motivation in PWS) |
| 7 | (McCleery and Nuechterlein, 2019) | Cognitive impairment in psychotic illness: prevalence, profile of impairment, developmental course, and treatment considerations | Is not a systematic review, rather it is a non-structured review |
| 8 | (Toh et al., 2018) | Impact of brain- derived neurotrophic factor genetic polymorphism on cognition: A systematic review | Not specifically in people with schizophrenia it is in HC and clinical samples in general |
| 9 | (van Kessel et al., 2020) | Psychotic depressive subtype and white mater hyperintensities do not predict cognitive side effects in ECT: A systematic review of pre-treatment predictors | Not in people with schizophrenia it is in people with unipolar, bipolar or psychotic depression |
| 10 | (Vita et al., 2018) | Psychopharmacological treatment of cognitive deficits in Schizophrenia and mood disorders | It is not a systematic review, but it is a kind of scoping review about treatment of cognitive impairment |
| 11 | (Wearne and Cornish, 2018) | A Comparison of Methamphetamine-Induced  Psychosis and Schizophrenia: A  Review of Positive, Negative, and Cognitive Symptomatology | It is a systematic review, but it is not specific to CIPWS and doesn’t address prevalence or factor associated with CIPWS; it compares symptoms among schizophrenic and meta amphetamine induced psychosis |
| **Backward search for articles included on the updated search** | | | |
| 1 | (Bora, 2017) | Relationship between insight and theory of mind in schizophrenia: A meta-analysis | It is a systematic review, but it does not address one full domain, it is a metanalysis of relationship between ToM and insight in PWS, |
| 2 | (Gibson et al., 2016) | Trauma and the psychosis spectrum: A review of symptom specificity and explanatory mechanisms | It is a systematic review, but not specific to CIPWS |
| 3 | (Green et al., 2019) | Non social and social cognition in schizophrenia: current evidence and future directions | Not systematic review, it is an overview (not systematically structured) of cognition in schizophrenia |
| 4 | (Pinkham, 2014) | Social Cognition in Schizophrenia | Not systematic review, it is an overview (not systematically structured) of social cognition in schizophrenia |
| 5 | (Brüne, 2005) | ‘‘Theory of Mind’’ in Schizophrenia: A Review of the Literature | It is a systematic review, but it does not address one full domain, it is a review of Theory of Mind in PWS |
| 6 | (Brunet-Gouet and Decety, 2006) | Social brain dysfunctions in schizophrenia: A review of neuroimaging studies | It is not a systematic review; it is a review of the structural changes associated with social cognition |
| 7 | (Harrington et al., 2005) | Theory of mind in schizophrenia: A critical review | It is a systematic review, but it does not address one full domain, it is a review of Theory of Mind in PWS |
| 8 | (Lewandowski et al., 2011) | Evolution of neuropsychological dysfunction during the course of schizophrenia and bipolar disorder | It doesn’t address concept of interest. In addition, it is a kind of review one data base is searched but results are not presented systematically |
| 9 | (Pinkham et al., 2003) | Implications for the Neural Basis of Social Cognition for the Study of Schizophrenia | It does not address concept of interest. In addition, it is a kind of review on which results are not presented systematically |
| 10 | (Van Overwalle, 2009) | Social Cognition and the Brain: A Meta-Analysis | It does not address concept of interest |
| 11 | (Mintz et al., 2003) | Insight in schizophrenia: a meta-analysis | It is not about CIPWS it is a systematic review of insight in schizophrenia in general not specific to CIPWS |
| **During forward search** | | | |
| 1 | (Aboulafia‐Brakha et al., 2011) | Theory of mind tasks and executive functions: | It is not in PWS, it is about cognitive impairment in PW acquired neurological disorders |
| 2 | (Ang and Pridmore, 2009) | Theory of mind and psychiatry: an introduction | Is not a systematic review, rather it is a non-structured review |
| 3 | (Baier, 2010) | Insight in Schizophrenia: A Review | Is not a systematic review, rather it is a non-structured review and not specific to CIPWS |
| 4 | (Barkl et al., 2014) | Facial emotion identification in early-onset and first-episode psychosis: A systematic review with meta-analysis | It is a systematic review, but it does not address one full domain, it is a review of Facial emotion identification |
| 5 | (Bickel et al., 2012) | Are executive function and impulsivity antipodes? A conceptual reconstruction with special reference to addiction | Is not a systematic review, rather it is a non-structured review and not specific to CIPWS |
| 6 | (Bora, 2009) | Theory of Mind in Schizophrenia Spectrum Disorders | Is not a systematic review, rather it is a non-structured review and does not address one full domain of CIPWS |
| 7 | (Bora et al., 2009b) | Theory of mind impairment: a distinct trait-marker for schizophrenia spectrum disorders and bipolar disorder? | It is a systematic review, but it does not address one full domain, it is a review of Theory of mind, and also it doesn’t address the concept of interest prevalence or associated factor |
| 8 | (Bortolato et al., 2015) | Cognitive dysfunction in bipolar disorder and schizophrenia: a systematic review of meta-analyses | It is a systematic review of meta-analyses |
| 9 | (Bosia et al., 2012) | Neurofunctional Correlates of Theory of Mind Deficits in Schizophrenia | Is not a systematic review, rather it is a non-structured review, does not address one full domain of CIPWS, and does not address the concept of interest |
| 10 | (Buck et al., 2020) | Hostile attribution bias in schizophrenia-spectrum  disorders: narrative review of the literature and persisting questions | It is a systematic review, but it does not address one full domain, it is a review of attribution bias a sub domain of SC |
| 11 | (Buck et al., 2016) | Revisiting the validity of measures of social cognitive bias in schizophrenia: Additional results from the Social Cognition Psychometric Evaluation (SCOPE) study | Is not a systematic review, rather it is a validation study and does not address the concept of interest |
| 12 | (Carbon and Correll, 2014) | Thinking and Acting Beyond the Positive: The Role of the Cognitive and Negative Symptoms in Schizophrenia | Is not a systematic review, rather it is an overview of negative and cognitive symptoms in PWS |
| 13 | (Chun et al., 2020) | Associations of psychotic-like experiences, related symptoms, and working memory with functioning | Is not a systematic review, rather it is a cross-sectional study |
| 14 | (Chung et al., 2016) | Beta-amyloid burden is not associated with cognitive impairment in schizophrenia: A systematic review | It doesn’t address concept of interest |
| 15 | (Chung et al., 2014) | A Meta-Analysis of Mentalizing Impairments in Adults With Schizophrenia and Autism Spectrum Disorder | It is a systematic review, but it does not address one full domain, it is a review of mentalizing impairments |
| 16 | (Corbera et al., 2013) | Factor Structure of Social Cognition in Schizophrenia: Is Empathy Preserved? | Is not a systematic review, rather it is a validation study |
| 17 | (Dimopoulou et al., 2017) | The Clinical Role of Theory of Mind in Schizophrenia: A Comprehensive Review | Is not a systematic review, rather it is a non-structured review, does not address one full domain of CIPWS, and does not address the concept of interest |
| 18 | (Dutt et al., 2015) | Exploring neural dysfunction in ‘clinical high risk’ for psychosis: A quantitative review of fMRI studies | It is a systematic review but not in PWS |
| 19 | (Fairfield et al., 2015) | Emotional Meta-Memories: A Review | It is not a systematic review it is a kind of overview; it doesn’t address CIPWS; and it is not in PWS |
| 20 | (Fernández-Sotos et al., 2018) | Pharmacological interventions in social cognition deficits: A systematic mapping review | It is a systematic review, but it is not specific to PWS it is a systematic review of SC in general (in any disorders) |
| 21 | (Fernandez-Sotos et al., 2019) | Social cognition remediation interventions: A systematic mapping review | It is a systematic review, but it is not specific to PWS it is a systematic review of SC in general (in any disorders) |
| 22 | (Ferrer et al., 2020) | Hypothalamic-pituitary-adrenal axis-related genes and cognition in major mood disorders and schizophrenia: a systematic review | It doesn’t address concept of interest |
| 23 | (Fiszdon and Reddy, 2012) | Review of social cognitive treatments for psychosis | It is not a systematic review, but it is an overview of SC treatment |
| 24 | (Green et al., 2013) | Has the Generalized Deficit Become the Generalized Criticism? | It is not a systematic review, but it is an editorial |
| 25 | (Harvey et al., 2019) | Prediction of disability in schizophrenia: Symptoms, cognition, and self-assessment | It is not a systematic review, but it is a kind of overview |
| 26 | (Jha, 2012) | Theory of Mind Deficit in Schizophrenia and Associated Cognitive Functions | It is not a systematic review; it is a cross-sectional study |
| 27 | (Joseph et al., 2015) | Insight in Schizophrenia: Relationship to Positive, Negative and Neurocognitive Dimensions | It is not a systematic review; it is a kind of overview of literatures about insight in schizophrenia |
| 28 | (Kar and Jain, 2016) | Current understandings about cognition and the neurobiological correlates in schizophrenia | It is not a systematic review; it is a kind of overview of literatures about cognition in schizophrenia |
| 29 | (Kluwe-Schiavon et al., 2013) | Executive functions rehabilitation for schizophrenia: A critical systematic review | It is a systematic review, but magnitude or associated factors are not addressed |
| 30 | (Kurtz et al., 2016) | Comprehensive treatments for social cognitive deficits in schizophrenia: A critical review and effect-size analysis of controlled studies | It is a systematic review, but magnitude or associated factors are not addressed |
| 31 | (Lee et al., 2015) | Social cognitive functioning in prodromal psychosis: A meta-analysis | Is a systematic review but it is not in PWS it is in individuals at clinical high risk |
| 32 | (Li et al., 2018) | Erythropoietin for Cognitive Deficits Associated with Schizophrenia, Bipolar Disorder, and Major Depression: A Systematic Review | It is a systematic review, but magnitude or associated factors are not addressed |
| 33 | (Madeira et al., 2016) | Social Cognition, Negative Symptoms and Psychosocial Functioning in Schizophrenia | It is not a systematic review; it is a kind of unstructured literatures about cognition in schizophrenia |
| 34 | (Mallet et al., 2020) | Polygenic Risk Scores Shed Light on the Relationship between Schizophrenia and Cognitive Functioning: Review and Meta-Analysis | It doesn’t address concept of interest |
| 35 | (Martin et al., 2015) | Social functioning and age across affective and non-affective psychoses | Is a not systematic review but it is a cross-sectional study; and does not address CIPWS; it addresses functioning. |
| 36 | (Matheson et al., 2014) | How much do we know about schizophrenia and how well do we know it? Evidence from the Schizophrenia Library | It is not a systematic review, rather it is a systematic review of systematic review; and it is not about CIPWS |
| 37 | (McCleery et al., 2015) | Meta-Analysis of Face Processing Event-Related Potentials in Schizophrenia | It is a systematic review, however the concept of interest is not addressed |
| 38 | (Misiak et al., 2018) | Cytokines and C-reactive protein alterations with respect to cognitive impairment in schizophrenia and bipolar disorder: A systematic review | It is a systematic review; however, the concept of interest is not addressed |
| 39 | (Mitchell and Young, 2016) | Theory of Mind in Bipolar Disorder, with Comparison to the Impairments Observed in Schizophrenia | It is not a systematic review; it is a kind of unstructured review and it doesn’t address one full domain of cognition |
| 40 | (Mortimer, 2008) | The neuropsychology of schizophrenia | It is not a systematic review; it is a kind of unstructured review |
| 41 | (Murri et al., 2015) | Is good insight associated with depression among patients with  schizophrenia? Systematic review and meta-analysis | It is a systematic review, but it is not about CIPWS |
| 42 | (Olagunju et al., 2019) | Long-acting atypical antipsychotics in schizophrenia: A systematic review and meta-analyses of effects on functional outcome | It is a systematic review, but it is not about CIPWS |
| 43 | (Palmer et al., 2015) | The relationship between cognitive insight and depression in psychosis and schizophrenia: A review and meta-analysis | It is a systematic review, but it is not about CIPWS |
| 44 | (Potvin et al., 2019) | A Functional Neuroimaging Meta-Analysis of Self-Related Processing in Schizophrenia | It is a systematic review; however the concept of interest is not addressed |
| 45 | (Smeland and Andreassen, 2018) | How can genetics help understand the relationship between cognitive dysfunction and schizophrenia? | It is not a systematic review; it is a kind of unstructured review |
| 46 | (Van Camp et al., 2017) | Cognitive insight: A systematic review | It is not a systematic review; it is a kind of unstructured review; and it is not about CIPWS |
| 47 | (Vaskinn and Horan, 2020) | Social Cognition and Schizophrenia: Unresolved Issues and New Challenges in a Maturing Field of Research | It is not a systematic review; it is a kind of unstructured review |
| 48 | (Zai et al., 2017) | A review of molecular genetic studies of neurocognitive deficits in schizophrenia | It is a systematic review; however, the concept of interest is not addressed |

**References**

ABOULAFIA‐BRAKHA, T., CHRISTE, B., MARTORY, M. D. & ANNONI, J. M. 2011. Theory of mind tasks and executive functions: a systematic review of group studies in neurology. *Journal of Neuropsychology,* 5**,** 39-55.

ACUNA-VARGAS, S. & THIBAUT, F. 2019. Cognition in psychiatry. *Dialogues in Clinical Neuroscience,* 21**,** 223-224.

AHMED, A. O. & BHAT, I. A. 2014. Psychopharmacological treatment of neurocognitive deficits in people with schizophrenia: a review of old and new targets. *CNS drugs,* 28**,** 301-318.

AHMED, A. O., FRIDBERG, D., HANNA, M. & BUCKLEY, P. F. 2013. Brain-Derived Neurotrophic Factor and neurocognitive profiles in the psychosis spectrum: findings in bipolar disorder and schizophrenia. *Bipolar Disorders: Symptoms, Management, and Risk Factors.* Nova Science Publishers, New York.

AHMED, A. O., MANTINI, A. M., FRIDBERG, D. J. & BUCKLEY, P. F. 2015. Brain-derived neurotrophic factor (BDNF) and neurocognitive deficits in people with schizophrenia: a meta-analysis. *Psychiatry Res,* 226**,** 1-13.

ALLEN, D. N., STRAUSS, G. P., DONOHUE, B. & VAN KAMMEN, D. P. 2007. Factor analytic support for social cognition as a separable cognitive domain in schizophrenia. *Schizophrenia research,* 93**,** 325-333.

ANG, G. K. & PRIDMORE, S. 2009. Theory of mind and psychiatry: an introduction. *Australasian Psychiatry,* 17**,** 117-122.

ANTONOVA, E., SHARMA, T., MORRIS, R. & KUMARI, V. 2004. The relationship between brain structure and neurocognition in schizophrenia: a selective review. *Schizophrenia research,* 70**,** 117-145.

ARMANDO, M., PAPALEO, F. & VICARI, S. 2012. COMT implication in cognitive and psychiatric symptoms in chromosome 22q11 microdeletion syndrome: a selective review. *CNS Neurol Disord Drug Targets,* 11**,** 273-81.

ARMANDO, M., PONTILLO, M., DE CRESCENZO, F., CORREALE, C., DE SIMONI, E., PAPALEO, F., SABA, R. & VICARI, S. 2013. An overview of correlations between schizophrenia and 22q11.2 deletion syndrome. *Clinical Neuropsychiatry: Journal of Treatment Evaluation,* 10**,** 3-10.

AYLWARD, E., WALKER, E. & BETTES, B. 1984. Intelligence in schizophrenia: meta-analysis of the research. *Schizophrenia bulletin,* 10**,** 430.

BAIER, M. 2010. Insight in schizophrenia: a review. *Current psychiatry reports,* 12**,** 356-361.

BARKL, S. J., LAH, S., HARRIS, A. W. & WILLIAMS, L. M. 2014. Facial emotion identification in early-onset and first-episode psychosis: a systematic review with meta-analysis. *Schizophrenia research,* 159**,** 62-69.

BHATTACHARYA, K. 2015. Cognitive function in schizophrenia: A review. *African Journal of Psychiatry (South Africa),* 18 (1) (no pagination).

BICKEL, W. K., JARMOLOWICZ, D. P., MUELLER, E. T., GATCHALIAN, K. M. & MCCLURE, S. M. 2012. Are executive function and impulsivity antipodes? A conceptual reconstruction with special reference to addiction. *Psychopharmacology,* 221**,** 361-387.

BILLEKE, P. & ABOITIZ, F. 2013. Social cognition in schizophrenia: From social stimuli processing to social engagement. *Frontiers in Psychiatry,* 4 (FEB) (no pagination).

BORA, E. 2009. Theory of Mind in Schizophrenia Spectrum Disorders. *Turkish Journal of Psychiatry,* 20.

BORA, E. 2015. Developmental trajectory of cognitive impairment in bipolar disorder: comparison with schizophrenia. *Eur Neuropsychopharmacol,* 25**,** 158-68.

BORA, E. 2017. Relationship between insight and theory of mind in schizophrenia: a meta-analysis. *Schizophrenia Research,* 190**,** 11-17.

BORA, E., YUCEL, M. & PANTELIS, C. 2009a. Theory of mind impairment in schizophrenia: Meta-analysis. *Schizophrenia Research,* 109**,** 1-9.

BORA, E., YÜCEL, M. & PANTELIS, C. 2009b. Theory of mind impairment: a distinct trait‐marker for schizophrenia spectrum disorders and bipolar disorder? *Acta Psychiatrica Scandinavica,* 120**,** 253-264.

BORTOLATO, B., MISKOWIAK, K. W., KÖHLER, C. A., VIETA, E. & CARVALHO, A. F. 2015. Cognitive dysfunction in bipolar disorder and schizophrenia: a systematic review of meta-analyses. *Neuropsychiatric disease and treatment,* 11**,** 3111.

BOSIA, M., RICCABONI, R. & POLETTI, S. 2012. Neurofunctional correlates of theory of mind deficits in schizophrenia. *Current topics in medicinal chemistry,* 12**,** 2284-2302.

BOWIE, C. R. & HARVEY, P. D. 2006. Cognitive deficits and functional outcome in schizophrenia. *Neuropsychiatric Disease and Treatment,* 2**,** 531-536.

BOZIKAS, V. P., KOSMIDIS, M. H. & KARAVATOS, A. 2004. Study of cognitive function in patients with schizophrenia. *Hellenic Journal of Psychology,* 1**,** 306-325.

BRÜNE, M. 2005. “Theory of mind” in schizophrenia: a review of the literature. *Schizophrenia Bulletin,* 31**,** 21-42.

BRUNET-GOUET, E. & DECETY, J. 2006. Social brain dysfunctions in schizophrenia: a review of neuroimaging studies. *Psychiatry Research: Neuroimaging,* 148**,** 75-92.

BUCK, B., BROWNE, J., GAGEN, E. C. & PENN, D. L. 2020. Hostile attribution bias in schizophrenia-spectrum disorders: narrative review of the literature and persisting questions. *Journal of Mental Health***,** 1-18.

BUCK, B. E., PINKHAM, A. E., HARVEY, P. D. & PENN, D. L. 2016. Revisiting the validity of measures of social cognitive bias in schizophrenia: Additional results from the Social Cognition Psychometric Evaluation (SCOPE) study. *British Journal of Clinical Psychology,* 55**,** 441-454.

BURNS, T. & PATRICK, D. 2007. Social functioning as an outcome measure in schizophrenia studies. *Acta Psychiatrica Scandinavica,* 116**,** 403-418.

CALAFATO, M. S. & BRAMON, E. 2019. The interplay between genetics, cognition and schizophrenia. *Brain: A Journal of Neurology,* 142**,** 236-238.

CANTOR-GRAAE, E., NORDSTRÖM, L. & MCNEIL, T. 2001. Substance abuse in schizophrenia: a review of the literature and a study of correlates in Sweden. *Schizophrenia research,* 48**,** 69-82.

CARBON, M. & CORRELL, C. U. 2014. Thinking and acting beyond the positive: the role of the cognitive and negative symptoms in schizophrenia. *CNS spectrums,* 19**,** 35-53.

CHUN, C. A., COOPER, S. & ELLMAN, L. M. 2020. Associations of psychotic-like experiences, related symptoms, and working memory with functioning. *European Psychiatry,* 63.

CHUNG, J. K., NAKAJIMA, S., PLITMAN, E., IWATA, Y., UY, D., GERRETSEN, P., CARAVAGGIO, F., CHAKRAVARTY, M. M. & GRAFF-GUERRERO, A. 2016. Β-Amyloid Burden is Not Associated with Cognitive Impairment in Schizophrenia: A Systematic Review. *The American Journal of Geriatric Psychiatry,* 24**,** 923-939.

CHUNG, Y. S., BARCH, D. & STRUBE, M. 2014. A meta-analysis of mentalizing impairments in adults with schizophrenia and autism spectrum disorder. *Schizophrenia bulletin,* 40**,** 602-616.

CORBERA, S., WEXLER, B. E., IKEZAWA, S. & BELL, M. D. 2013. Factor structure of social cognition in schizophrenia: is empathy preserved? *Schizophrenia research and treatment,* 2013.

COTTER, J., GRANGER, K., BACKX, R., HOBBS, M., LOOI, C. Y. & BARNETT, J. H. 2018. Social cognitive dysfunction as a clinical marker: A systematic review of meta-analyses across 30 clinical conditions. *Neuroscience and Biobehavioral Reviews,* 84**,** 92-99.

DABAN, C., AMADO, I., BAYLÉ, F., GUT, A., WILLARD, D., BOURDEL, M.-C., LOO, H., OLIÉ, J.-P., MILLET, B. & KREBS, M.-O. 2002. Correlation between clinical syndromes and neuropsychological tasks in unmedicated patients with recent onset schizophrenia. *Psychiatry Research,* 113**,** 83-92.

DICKSON, H., LAURENS, K. R., CULLEN, A. E. & HODGINS, S. 2012. Meta-analyses of cognitive and motor function in youth aged 16 years and younger who subsequently develop schizophrenia. *Psychological medicine,* 42**,** 743-755.

DIMOPOULOU, T., TARAZI, F. I. & TSAPAKIS, E. M. 2017. The Clinical Role of Theory of Mind in Schizophrenia: A Comprehensive Review.

DUTT, A., TSENG, H.-H., FONVILLE, L., DRAKESMITH, M., SU, L., EVANS, J., ZAMMIT, S., JONES, D., LEWIS, G. & DAVID, A. S. 2015. Exploring neural dysfunction in ‘clinical high risk’for psychosis: a quantitative review of fMRI studies. *Journal of psychiatric research,* 61**,** 122-134.

ELIE, D., POIRIER, M., CHIANETTA, J., DURAND, M., GRÉGOIRE, C. & GRIGNON, S. 2010. Cognitive effects of antipsychotic dosage and polypharmacy: a study with the BACS in patients with schizophrenia and schizoaffective disorder. *Journal of Psychopharmacology,* 24**,** 1037-1044.

FAIRFIELD, B., MAMMARELLA, N., PALUMBO, R. & DI DOMENICO, A. 2015. Emotional meta-memories: a review. *Brain sciences,* 5**,** 509-520.

FERNÁNDEZ-SOTOS, P., NAVARRO, E., TORIO, I., DOMPABLO, M., FERNÁNDEZ-CABALLERO, A. & RODRIGUEZ-JIMENEZ, R. 2018. Pharmacological interventions in social cognition deficits: a systematic mapping review. *Psychiatry Research,* 270**,** 57-67.

FERNANDEZ-SOTOS, P., TORIO, I., FERNANDEZ-CABALLERO, A., NAVARRO, E., GONZALEZ, P., DOMPABLO, M. & RODRIGUEZ-JIMENEZ, R. 2019. Social cognition remediation interventions: A systematic mapping review. *PloS one,* 14**,** e0218720.

FERRER, A., LABAD, J., SALVAT-PUJOL, N., MONREAL, J. A., URRETAVIZCAYA, M., CRESPO, J. M., MENCHÓN, J. M., PALAO, D. & SORIA, V. 2020. Hypothalamic-pituitary-adrenal axis-related genes and cognition in major mood disorders and schizophrenia: a systematic review. *Progress in Neuro-Psychopharmacology and Biological Psychiatry***,** 109929.

FISZDON, J. M. & REDDY, L. F. 2012. Review of social cognitive treatments for psychosis. *Clinical Psychology Review,* 32**,** 724-740.

FRANGOU, S. 2013. Neurocognition in early-onset schizophrenia. *Child and Adolescent Psychiatric Clinics of North America,* 22**,** 715-726.

FUSAR-POLI, P., DESTE, G., SMIESKOVA, R., BARLATI, S., YUNG, A. R., HOWES, O., STIEGLITZ, R.-D., VITA, A., MCGUIRE, P. & BORGWARDT, S. 2012. Cognitive functioning in prodromal psychosis: A meta-analysis. *JAMA Psychiatry,* 69**,** 562-571.

GIBSON, L. E., ALLOY, L. B. & ELLMAN, L. M. 2016. Trauma and the psychosis spectrum: a review of symptom specificity and explanatory mechanisms. *Clinical Psychology Review,* 49**,** 92-105.

GREEN, M. F. 2016. Impact of cognitive and social cognitive impairment on functional outcomes in patients with schizophrenia. *The Journal of clinical psychiatry,* 77**,** 8-11.

GREEN, M. F., BEARDEN, C. E., CANNON, T. D., FISKE, A. P., HELLEMANN, G. S., HORAN, W. P., KEE, K., KERN, R. S., LEE, J. & SERGI, M. J. 2011. Social cognition in schizophrenia, part 1: performance across phase of illness. *Schizophrenia bulletin,* 38**,** 854-864.

GREEN, M. F., HORAN, W. P. & LEE, J. 2015. Social cognition in schizophrenia. *Nature Reviews Neuroscience,* 16**,** 620.

GREEN, M. F., HORAN, W. P. & LEE, J. 2019. Nonsocial and social cognition in schizophrenia: current evidence and future directions. *World Psychiatry,* 18**,** 146-161.

GREEN, M. F., HORAN, W. P. & SUGAR, C. A. 2013. Has the generalized deficit become the generalized criticism? *Schizophrenia bulletin,* 39**,** 257-262.

GREEN, M. F., KERN, R. S., BRAFF, D. L. & MINTZ, J. 2000. Neurocognitive deficits and functional outcome in schizophrenia: are we measuring the "right stuff"? *Schizophr Bull,* 26**,** 119-36.

GREEN, M. F., KERN, R. S. & HEATON, R. K. 2004. Longitudinal studies of cognition and functional outcome in schizophrenia: implications for MATRICS. *Schizophrenia research,* 72**,** 41-51.

GRUBE, B. S., BILDER, R. M. & GOLDMAN, R. S. 1998. Meta-analysis of symptom factors in schizophrenia. *Schizophrenia Research,* 31**,** 113-120.

HAHN, S. R. 2000. Cognitive decline in late-life schizophrenia and alzheimer's disease: A longitudinal comparison of performance on the Mini-Mental Status Exam. *Dissertation Abstracts International: Section B: The Sciences and Engineering,* 60**,** 4223.

HAMILTON, G. R. 2019. The utility of neurocognitive profiles in diagnosing comorbid schizophrenia and intellectual developmental disorder. *Dissertation Abstracts International: Section B: The Sciences and Engineering,* 80.

HARRINGTON, L., SIEGERT, R. & MCCLURE, J. 2005. Theory of mind in schizophrenia: a critical review. *Cognitive neuropsychiatry,* 10**,** 249-286.

HARVEY, P. D. 2001. Cognitive impairment in elderly patients with schizophrenia: Age related changes. *International Journal of Geriatric Psychiatry,* 16**,** S78-S85.

HARVEY, P. D. 2005. Cognition and Function in Older Patients With Schizophrenia. *Harvey, Philip D (2005) Schizophrenia in late life: Aging effects on symptoms and course of illness (pp 53-70) v, 219 pp Washington, DC, US: American Psychological Association; US***,** 53-70.

HARVEY, P. D. & KEEFE, R. S. 2001. Studies of cognitive change in patients with schizophrenia following novel antipsychotic treatment. *American Journal of Psychiatry,* 158**,** 176-184.

HARVEY, P. D., LOEWENSTEIN, D. A. & CZAJA, S. J. 2013. Hospitalization and psychosis: Influences on the course of cognition and everyday functioning in people with schizophrenia. *Neurobiology of Disease,* 53**,** 18-25.

HARVEY, P. D., REICHENBERG, A. & BOWIE, C. R. 2006. Cognition and aging in psychopathology: Focus on schizophrenia and depression. *Annual Review of Clinical Psychology,* 2**,** 389-409.

HARVEY, P. D., STRASSNIG, M. T. & SILBERSTEIN, J. 2019. Prediction of disability in schizophrenia: Symptoms, cognition, and self-assessment. *Journal of Experimental Psychopathology,* 10**,** 2043808719865693.

HEMSLEY, D. R. 1993. A simple (or simplistic?) cognitive model for schizophrenia. *Behaviour research and therapy,* 31**,** 633-645.

HILL, S. K., SCHUEPBACH, D., HERBENER, E. S., KESHAVAN, M. S. & SWEENEY, J. A. 2004. Pretreatment and longitudinal studies of neuropsychological deficits in antipsychotic-naıve patients with schizophrenia. *Schizophrenia research,* 68**,** 49-63.

HOFF, A. L. & KREMEN, W. S. 2003. Neuropsychology in schizophrenia: an update. *Current Opinion in Psychiatry,* 16**,** 149-155.

HORI, H., NOGUCHI, H., HASHIMOTO, R., NAKABAYASHI, T., OMORI, M., TAKAHASHI, S., TSUKUE, R., ANAMI, K., HIRABAYASHI, N. & HARADA, S. 2006. Antipsychotic medication and cognitive function in schizophrenia. *Schizophrenia research,* 86**,** 138-146.

JAASKELAINEN, I. P., STENBERG, J.-H., HAKKINEN-RIHU, P. & ROYKS, R. 1997. Cognitive deficits in schizophrenia. *Psykologia,* 32**,** 360-365.

JANOVIC, S., BAJS, M., BAJS, M. & OREVIC, V. 2006. Cognitive impairment in schizophrenia. *Socijalna Psihijatrija,* 34**,** 175-182.

JELLINGER, K. 2001. Dementia as a complication of schizophrenia. *Journal of Neurology, Neurosurgery & Psychiatry,* 71**,** 707-708.

JHA, M. 2012. Theory of mind deficit in schizophrenia and associated cognitive functions. *Psychological Studies,* 57**,** 283-291.

JOSEPH, B., NARAYANASWAMY, J. C. & VENKATASUBRAMANIAN, G. 2015. Insight in schizophrenia: relationship to positive, negative and neurocognitive dimensions. *Indian journal of psychological medicine,* 37**,** 5.

KALADJIAN, A., AZORIN, J., POMIETTO, P., CORREARD, N., BELZEAUX, R. & ADIDA, M. 2012. Schizophrenia and/or bipolar disorder: The neurocognitive endophenotypes. *L'Encephale: Revue de psychiatrie clinique biologique et therapeutique,* 38**,** S81-S84.

KALIN, N. H. 2020. Psychotic experiences, cognitive decline, and genetic vulnerabilities in relation to developing psychotic disorders. *American Journal of Psychiatry,* 177**,** 279-281.

KAMBEITZ, J. P., BHATTACHARYYA, S., KAMBEITZ-ILANKOVIC, L. M., VALLI, I., COLLIER, D. A. & MCGUIRE, P. 2012. Effect of BDNF val66met polymorphism on declarative memory and its neural substrate: a meta-analysis. *Neuroscience & Biobehavioral Reviews,* 36**,** 2165-2177.

KAR, S. K. & JAIN, M. 2016. Current understandings about cognition and the neurobiological correlates in schizophrenia. *Journal of neurosciences in rural practice,* 7**,** 412.

KERI, S. & JANKA, Z. 2003. Cognitive dysfunctions in schizophrenia: Are they endophenotypes? *Psychiatria Hungarica,* 18**,** 373-380.

KERI, S. & JANKA, Z. 2004. Critical evaluation of cognitive dysfunctions as endophenotypes of schizophrenia. *Acta Psychiatr Scand,* 110**,** 83-91.

KIMOTO, S., MAKINODAN, M. & KISHIMOTO, T. 2019. Neurobiology and treatment of social cognition in schizophrenia: Bridging the bed-bench gap. *Neurobiology of Disease,* 131 (no pagination).

KLUWE-SCHIAVON, B., SANVICENTE-VIEIRA, B., KRISTENSEN, C. & GRASSI-OLIVEIRA, R. 2013. Executive functions rehabilitation for schizophrenia: a critical systematic review. *Journal of Psychiatric Research,* 47**,** 91-104.

KOMLOSI, S., CZOBOR, P., BALINT, S. & BITTER, I. 2008. [The relationship between cognition and functional outcome in schizophrenia]. *Psychiatr Hung,* 23**,** 166-76.

KOREN, D. & HARVEY, P. D. 2006. Closing the gap between cognitive performance and real-world functional outcome in schizophrenia: The importance of metacognition. *Current Psychiatry Reviews,* 2**,** 189-198.

KREMEN, W. S., SEIDMAN, L. J., PEPPLE, J. R., LYONS, M. J., TSUANG, M. T. & FARAONE, S. V. 1994. Neuropsychological risk indicators for schizophrenia: a review of family studies. *Schizophrenia Bulletin,* 20**,** 103-119.

KRIVOY, A., FISCHEL, T. & WEIZMAN, A. 2012. [The cognitive deficit in schizophrenia]. *Harefuah,* 151**,** 277-80, 319.

KURTZ, M. M. 2005. Neurocognitive impairment across the lifespan in schizophrenia: an update. *Schizophrenia research,* 74**,** 15-26.

KURTZ, M. M. 2012. Neurocognition and functional outcome in schizophrenia. *Marcopulos, Bernice A [Ed]; Kurtz, Matthew M [Ed] (2012) Clinical neuropsychological foundations of schizophrenia (pp 81-101) xvi, 351 pp New York, NY, US: Psychology Press; US***,** 81-101.

KURTZ, M. M., GAGEN, E., ROCHA, N. B., MACHADO, S. & PENN, D. L. 2016. Comprehensive treatments for social cognitive deficits in schizophrenia: A critical review and effect-size analysis of controlled studies. *Clinical psychology review,* 43**,** 80-89.

KURTZ, M. M. & MARCOPULOS, B. A. 2012. Cognition in schizophrenia. *Marcopulos, Bernice A [Ed]; Kurtz, Matthew M [Ed] (2012) Clinical neuropsychological foundations of schizophrenia (pp 1-25) xvi, 351 pp New York, NY, US: Psychology Press; US***,** 1-25.

LANDGRAF, S., AMADO, I., BERTHOZ, A., MARIE, O. & VAN DER MEERA, E. 2012. Cognitive identity in schizophrenia: vision, space, and body perception from prodrome to syndrome. *Current Psychiatry Reviews,* 8**,** 119-139.

LARGE, M., SHARMA, S., COMPTON, M. T., SLADE, T. & NIELSSEN, O. 2011. Cannabis use and earlier onset of psychosis: a systematic meta-analysis. *Archives of general psychiatry,* 68**,** 555-561.

LAWS, K. R. 1999. A meta-analytic review of Wisconsin Card Sort studies in schizophrenia: general intellectual deficit in disguise? *Cognitive Neuropsychiatry,* 4**,** 1-30.

LEE, T. Y., HONG, S. B., SHIN, N. Y. & KWON, J. S. 2015. Social cognitive functioning in prodromal psychosis: a meta-analysis. *Schizophrenia research,* 164**,** 28-34.

LENKA, A., HEGDE, S., ARUMUGHAM, S. S. & PAL, P. K. 2017. Pattern of cognitive impairment in patients with Parkinson's disease and psychosis: A critical review. *Parkinsonism Relat Disord,* 37**,** 11-18.

LEWANDOWSKI, K., COHEN, B. & ÖNGUR, D. 2011. Evolution of neuropsychological dysfunction during the course of schizophrenia and bipolar disorder. *Psychological medicine,* 41**,** 225.

LEWIS, D. A. & GLAUSIER, J. R. 2016. *Alterations in prefrontal cortical circuitry and cognitive dysfunction in schizophrenia*.

LI, X.-B., ZHENG, W., NING, Y.-P., CAI, D.-B., YANG, X.-H., UNGVARI, G. S., NG, C. H., WANG, C.-Y. & XIANG, Y.-T. 2018. Erythropoietin for cognitive deficits associated with schizophrenia, bipolar disorder, and major depression: a systematic review. *Pharmacopsychiatry,* 51**,** 100-104.

LINDENMAYER, J.-P., GROCHOWSKI, S. & HYMAN, R. B. 1995. Five factor model of schizophrenia: replication across samples. *Schizophrenia research,* 14**,** 229-234.

LOBERG, E.-M. & HUGDAHL, K. 2009. Cannabis use and cognition in schizophrenia. *Frontiers in Human Neuroscience Vol 3 2009, ArtID 53,* 3.

LUNGU, O., BARAKAT, M., LAVENTURE, S., DEBAS, K., PROULX, S., LUCK, D. & STIP, E. 2013. The incidence and nature of cerebellar findings in schizophrenia: a quantitative review of fMRI literature. *Schizophr Bull,* 39**,** 797-806.

LUTHER, L., FIRMIN, R. L., LYSAKER, P. H., MINOR, K. S. & SALYERS, M. P. 2018. A meta-analytic review of self-reported, clinician-rated, and performance-based motivation measures in schizophrenia: Are we measuring the same "stuff"? *Clinical Psychology Review,* 61**,** 24-37.

MADEIRA, N., CALDEIRA, S., BAJOUCO, M., PEREIRA, A. T., MARTINS, M. J. & MACEDO, A. 2016. Social Cognition, Negative Symptoms and Psychosocial Functioning in Schizophrenia.

MALLA, A., NORMAN, R., MANCHANDA, R. & TOWNSEND, L. 2002. Symptoms, cognition, treatment adherence and functional outcome in first-episode psychosis. *Psychological Medicine,* 32**,** 1109-1119.

MALLET, J., LE STRAT, Y., DUBERTRET, C. & GORWOOD, P. 2020. Polygenic Risk Scores Shed Light on the Relationship between Schizophrenia and Cognitive Functioning: Review and Meta-Analysis. *Journal of clinical medicine,* 9**,** 341.

MANDELMAN, S. D. & GRIGORENKO, E. L. 2012. BDNF Val66Met and cognition: all, none, or some? A meta‐analysis of the genetic association. *Genes, Brain and Behavior,* 11**,** 127-136.

MARTIN, E. A., ÖNGÜR, D., COHEN, B. M. & LEWANDOWSKI, K. E. 2015. Social functioning and age across affective and non-affective psychoses. *The Journal of nervous and mental disease,* 203**,** 37.

MATHESON, S., SHEPHERD, A. & CARR, V. 2014. How much do we know about schizophrenia and how well do we know it? Evidence from the Schizophrenia Library. *Psychological medicine,* 44**,** 3387.

MCCLEERY, A., LEE, J., JOSHI, A., WYNN, J. K., HELLEMANN, G. S. & GREEN, M. F. 2015. Meta-analysis of face processing event-related potentials in schizophrenia. *Biological psychiatry,* 77**,** 116-126.

MCCLEERY, A. & NUECHTERLEIN, K. H. 2019. Cognitive impairment in psychotic illness: prevalence, profile of impairment, developmental course, and treatment considerations *Dialogues Clin Neurosci,* 21**,** 239-248.

MCGURK, S. R. & MUESER, K. T. 2013. Cognition and work functioning in schizophrenia. *Harvey, Philip D [Ed] (2013) Cognitive impairment in schizophrenia: Characteristics, assessment and treatment (pp 98-109) xv, 324 pp New York, NY, US: Cambridge University Press; US***,** 98-109.

MINTZ, A. R., DOBSON, K. S. & ROMNEY, D. M. 2003. Insight in schizophrenia: a meta-analysis. *Schizophrenia research,* 61**,** 75-88.

MISIAK, B., STAŃCZYKIEWICZ, B., KOTOWICZ, K., RYBAKOWSKI, J. K., SAMOCHOWIEC, J. & FRYDECKA, D. 2018. Cytokines and C-reactive protein alterations with respect to cognitive impairment in schizophrenia and bipolar disorder: a systematic review. *Schizophrenia research,* 192**,** 16-29.

MITCHELL, R. L. & YOUNG, A. H. 2016. Theory of mind in bipolar disorder, with comparison to the impairments observed in schizophrenia. *Frontiers in psychiatry,* 6**,** 188.

MORITZ, S. & WOODWARD, T. S. 2006. Metacognitive control over false memories: A key determinant of delusional thinking. *Current Psychiatry Reports,* 8**,** 184-190.

MORTIMER, A. M. 2008. The neuropsychology of schizophrenia. *Psychiatry,* 7**,** 435-439.

MURRI, M. B., RESPINO, M., INNAMORATI, M., CERVETTI, A., CALCAGNO, P., POMPILI, M., LAMIS, D. A., GHIO, L. & AMORE, M. 2015. Is good insight associated with depression among patients with schizophrenia? Systematic review and meta-analysis. *Schizophrenia research,* 162**,** 234-247.

NESTOR, P. G., NIZNIKIEWICZ, M. & MCCARLEY, R. W. 2010. Distinct contribution of working memory and social comprehension failures in neuropsychological impairment in schizophrenia. *Journal of Nervous and Mental Disease,* 198**,** 206-212.

NIIZATO, K., GENDA, K., NAKAMURA, R., IRITANI, S. & IKEDA, K. 2001. Cognitive decline in schizophrenics with Alzheimer's disease: a mini-review of neuropsychological and neuropathological studies. *Progress in neuro-psychopharmacology & biological psychiatry*.

OHI, K., SUMIYOSHI, C., FUJINO, H., YASUDA, Y., YAMAMORI, H., FUJIMOTO, M., SUMIYOSHI, T. & HASHIMOTO, R. 2017. A brief assessment of intelligence decline in schizophrenia as represented by the difference between current and premorbid intellectual quotient. *Frontiers in Psychiatry Vol 8 2017, ArtID 293,* 8.

OKRUSZEK, L. & PILECKA, I. 2017. Biological motion processing in schizophrenia - Systematic review and meta-analysis. *Schizophr Res,* 190**,** 3-10.

OLAGUNJU, A. T., CLARK, S. R. & BAUNE, B. T. 2019. Long-acting atypical antipsychotics in schizophrenia: A systematic review and meta-analyses of effects on functional outcome. *Australian & New Zealand Journal of Psychiatry,* 53**,** 509-527.

PALMER, E. C., GILLEEN, J. & DAVID, A. S. 2015. The relationship between cognitive insight and depression in psychosis and schizophrenia: a review and meta-analysis. *Schizophrenia Research,* 166**,** 261-268.

PAPA, D. & BERSANI, G. 2007. Face, identity and emotion in schizophrenia: From perception to expression. [Italian]. *Italian Journal of Psychopathology,* 13**,** 14-25.

PINKHAM, A. E. 2014. Social cognition in schizophrenia. *The Journal of clinical psychiatry*.

PINKHAM, A. E., PENN, D. L., PERKINS, D. O. & LIEBERMAN, J. 2003. Implications for the neural basis of social cognition for the study of schizophrenia. *American Journal of Psychiatry,* 160**,** 815-824.

PISKULIC, D. & ADDINGTON, J. 2011. Social cognition and negative symptoms in psychosis. *Psychiatry Research,* 188**,** 283-285.

POTVIN, S., GAMACHE, L. & LUNGU, O. 2019. A functional neuroimaging meta-analysis of self-related processing in schizophrenia. *Frontiers in neurology,* 10**,** 990.

RAJAKUMAR, N. 2017. Prefrontal cortical abnormalities in cognitive deficits of schizophrenia. *Cechetto, David [Ed]; Weishaupt, Nina [Ed] (2017) The cerebral cortex in neurodegenerative and neuropsychiatric disorders: Experimental approaches to clinical issues (pp 277-287) xix, 320 pp San Diego, CA, US: Elsevier Academic Press; US***,** 277-287.

RAUCHER-CHENE, D., CUERVO-LOMBARD, C., BERA-POTELLE, C. & HAVET, J. 2011. Schizophrenia in the elderly: Clinical, cognitive and social features. *NPG Neurologie - Psychiatrie - Geriatrie,* 11**,** 157-165.

REICHENBERG, A. 2005. Cognitive impairment as a risk factor for psychosis. *Dialogues Clin Neurosci,* 7**,** 31-8.

RINGDAHL, E. N. 2015. Impaired theory of mind in psychotic and affective disorders. *Dissertation Abstracts International: Section B: The Sciences and Engineering,* 76**,** No Pagination Specified.

ROY, M. A. & DEVRIENDT, X. 1994. [Positive and negative symptoms in schizophrenia: a current overview]. *Can J Psychiatry,* 39**,** 407-14.

RUND, B. R. 1998. A review of longitudinal studies of cognitive functions in schizophrenia patients. *Schizophrenia bulletin,* 24**,** 425-435.

RUND, B. R. 2009. Is schizophrenia a neurodegenerative disorder? *Nordic journal of psychiatry,* 63**,** 196-201.

SAROLTA, K., PAL, C., SARA, B. & ISTVAN, B. 2008. The relationship between cognition and functional outcome in schizophrenia. *Psychiatria Hungarica,* 23**,** 166-176.

SCHIMMELMANN, B. G., CONUS, P., COTTON, S., MCGORRY, P. D. & LAMBERT, M. 2007. Pre-treatment, baseline, and outcome differences between early-onset and adult-onset psychosis in an epidemiological cohort of 636 first-episode patients. *Schizophrenia research,* 95**,** 1-8.

SCHMI, L., LASSE, M. & SCHRODER, J. 2011. Symptoms and cognition in geriatric schizophrenia. *Fortschritte der Neurologie, Psychiatrie,* 79**,** 267-276.

SCHNUR, P. & HOFFMAN, A. C. 2010. Nicotinic modulation of attentional deficits in schizophrenia. *Lubow, Robert E [Ed]; Weiner, Ina [Ed] (2010) Latent inhibition: Cognition, neuroscience and applications to schizophrenia (pp 477-499) xii, 561 pp New York, NY, US: Cambridge University Press; US***,** 477-499.

SCHÜRHOFF, F., KREBS, M. O., SZÖKE, A., LOZE, J. Y., GOLDBERGER, C., QUIGNON, V., TIGNOL, J., ROUILLON, F., LAPLANCHE, J. L. & LEBOYER, M. 2003. Apolipoprotein E in schizophrenia: A French association study and meta‐analysis. *American Journal of Medical Genetics Part B: Neuropsychiatric Genetics,* 119**,** 18-23.

SCHWARTZ, B. D., TOMLIN, H. R., EVANS, W. J. & ROSS, K. V. 2001. Neurophysiologic mechanisms of attention: a selective review of early information processing in schizophrenics. *Front Biosci,* 6**,** D120-D134.

SCOTT, J. C., WOODS, S. P., MATT, G. E., MEYER, R. A., HEATON, R. K., ATKINSON, J. H. & GRANT, I. 2007. Neurocognitive effects of methamphetamine: a critical review and meta-analysis. *Neuropsychology review,* 17**,** 275-297.

SEVY, S., BURDICK, K. E., VISWESWARAIAH, H., ABDELMESSIH, S., LUKIN, M., YECHIAM, E. & BECHARA, A. 2007. Iowa gambling task in schizophrenia: a review and new data in patients with schizophrenia and co-occurring cannabis use disorders. *Schizophrenia research,* 92**,** 74-84.

SMELAND, O. B. & ANDREASSEN, O. A. 2018. How can genetics help understand the relationship between cognitive dysfunction and schizophrenia? *Scandinavian journal of psychology,* 59**,** 26-31.

SONG, M. J., CHOI, H. I., JANG, S.-K., LEE, S.-H., IKEZAWA, S. & CHOI, K.-H. 2015. Theory of mind in Koreans with schizophrenia: A meta-analysis. *Psychiatry Research,* 229**,** 420-425.

SPENCER, B. W. J., SHIELDS, G., GERGEL, T., HOTOPF, M. & OWEN, G. S. 2017. Diversity or disarray? A systematic review of decision-making capacity for treatment and research in schizophrenia and other non-affective psychoses. *Psychol Med,* 47**,** 1906-1922.

SPRONG, M., SCHOTHORST, P., VOS, E., HOX, J. & VAN ENGELAND, H. 2007. Theory of mind in schizophrenia: meta-analysis. *Br J Psychiatry,* 191**,** 5-13.

STIP, E. 2006. [Cognition, schizophrenia and the effect of antipsychotics]. *Encephale,* 32**,** 341-50.

STRAUSS, E., SHERMAN, E. M. & SPREEN, O. 2006. *A compendium of neuropsychological tests: Administration, norms, and commentary*, American Chemical Society.

STUART, G., PANTELIS, C., KLIMIDIS, S. & MINAS, I. 1999. The three-syndrome model of schizophrenia: Meta-analysis of an artefact. *Schizophrenia Research,* 39**,** 233-242.

THOMPSON, A. D., BARTHOLOMEUSZ, C. & YUNG, A. R. 2011. Social cognition deficits and the ‘ultra high risk’for psychosis population: a review of literature. *Early intervention in psychiatry,* 5**,** 192-202.

TOH, Y. L., NG, T., TAN, M., TAN, A. & CHAN, A. 2018. Impact of brain-derived neurotrophic factor genetic polymorphism on cognition: A systematic review. *Brain and Behavior,* 8 (7) (no pagination).

TYBURSKI, E., SOKOLOWSKI, A., CHEC, M., PELKA-WYSIECKA, J. & SAMOCHOWIEC, A. 2015. Neuropsychological characteristics of verbal and non-verbal fluency in schizophrenia patients. *Arch Psychiatr Nurs,* 29**,** 33-8.

VAN CAMP, L., SABBE, B. & OLDENBURG, J. 2017. Cognitive insight: A systematic review. *Clinical psychology review,* 55**,** 12-24.

VAN KESSEL, M. A., VAN DER VLUGT, J. J. B., SPAANS, H.-P., MURRE, J. M. J. & VERWIJK, E. 2020. Psychotic depressive subtype and white mater hyperintensities do not predict cognitive side effects in ECT: A systematic review of pretreatment predictors. *Journal of Affective Disorders,* 272**,** 340-347.

VAN OVERWALLE, F. 2009. Social cognition and the brain: a meta‐analysis. *Human brain mapping,* 30**,** 829-858.

VAN WINKEL, R., STEFANIS, N. C. & MYIN-GERMEYS, I. 2008. Psychosocial stress and psychosis. A review of the neurobiological mechanisms and the evidence for gene-stress interaction. *Schizophrenia bulletin,* 34**,** 1095-1105.

VARESE, F. & BENTALL, R. P. 2011. The metacognitive beliefs account of hallucinatory experiences: A literature review and meta-analysis. *Clinical Psychology Review,* 31**,** 850-864.

VASKINN, A. & HORAN, W. P. 2020. Social Cognition and Schizophrenia: Unresolved Issues and New Challenges in a Maturing Field of Research. *Schizophrenia Bulletin,* 46**,** 464-470.

VENTURA, J., HELLEMANN, G. S., THAMES, A. D., KOELLNER, V. & NUECHTERLEIN, K. H. 2009. Symptoms as mediators of the relationship between neurocognition and functional outcome in schizophrenia: A meta-analysis. *Schizophrenia Research,* 113**,** 189-199.

VENTURA, J., THAMES, A. D., WOOD, R. C., GUZIK, L. H. & HELLEMANN, G. S. 2010. Disorganization and reality distortion in schizophrenia: A meta-analysis of the relationship between positive symptoms and neurocognitive deficits. *Schizophrenia Research,* 121**,** 1-14.

VENTURA, J., WOOD, R. C. & HELLEMANN, G. S. 2013a. Symptom domains and neurocognitive functioning can help differentiate social cognitive processes in schizophrenia: A meta-analysis. *Schizophrenia Bulletin,* 39**,** 102-111.

VENTURA, J., WOOD, R. C., JIMENEZ, A. M. & HELLEMANN, G. S. 2013b. Neurocognition and symptoms identify links between facial recognition and emotion processing in schizophrenia: meta-analytic findings. *Schizophr Res,* 151**,** 78-84.

VILLALTA-GIL, V., VILAPLANA, M., OCHOA, S., HARO, J. M., DOLZ, M., USALL, J., CERVILLA, J. & GROUP, N. 2006. Neurocognitive performance and negative symptoms: are they equal in explaining disability in schizophrenia outpatients? *Schizophrenia research,* 87**,** 246-253.

VITA, A., MUSSONI, C., DESTE, G., FERLENGHI, G., TURRINA, C. & VALSECCHI, P. 2018. Psychopharmacological treatment of cognitive deficits in Schizophrenia and mood disorders. *Journal of Psychopathology,* 24**,** 62-72.

VYAS, N. S., WANG, Y., VYAS, S. S., KILLACKEY, E., HAUGLAND, T.-A. & CHAN, R. C. 2017. Theory of mind in the early course of schizophrenia. *Current Psychiatry Reviews,* 13**,** 102-110.

WEARNE, T. A. & CORNISH, J. L. 2018. A comparison of methamphetamine-induced psychosis and schizophrenia: A review of positive, negative, and cognitive symptomatology. *Frontiers in Psychiatry,* 9 (OCT) (no pagination).

WEINBERGER, A. H., CREEDEN, C. L., SACCO, K. A. & GEORGE, T. P. 2007. Neurocognitive effects of nicotine and tobacco in individuals with schizophrenia. *Journal of Dual Diagnosis,* 3**,** 61-77.

WOLF, M. E., DEWOLFE, A. S. & MOSNAIM, A. D. 1991. The association of tardive dyskinesia with cognitive deficits: A review. *Research Communications in Psychology, Psychiatry & Behavior,* 16**,** 15-27.

ZAI, G., ROBBINS, T. W., SAHAKIAN, B. J. & KENNEDY, J. L. 2017. A review of molecular genetic studies of neurocognitive deficits in schizophrenia. *Neuroscience & Biobehavioral Reviews,* 72**,** 50-67.
